# Activated Peyer′s patch B cells sample antigen directly from M cells in the subepithelial dome

**DOI:** 10.1038/s41467-019-10144-w

**Published:** 2019-06-03

**Authors:** Rathan Joy Komban, Anneli Strömberg, Adi Biram, Jakob Cervin, Cristina Lebrero-Fernández, Neil Mabbott, Ulf Yrlid, Ziv Shulman, Mats Bemark, Nils Lycke

**Affiliations:** 1 Mucosal Immunobiology and Vaccine Center (MIVAC), Department of Microbiology and Immunology, Institute of Biomedicine, University of Gothenburg, Gothenburg, S405 30 Sweden; 2 Department of Immunology, The Weizmann Institute of Science, Rehovot, 76100 Israel; 3 The Roslin Institute, Edinburgh University, Edinburgh, EH25 9RG Scotland

**Keywords:** Antibodies, Antigen processing and presentation, B cells, Mucosal immunology

## Abstract

The germinal center (GC) reaction in Peyer′s patches (PP) requires continuous access to antigens, but how this is achieved is not known. Here we show that activated antigen-specific CCR6^+^CCR1^+^GL7^−^ B cells make close contact with M cells in the subepithelial dome (SED). Using in situ photoactivation analysis of antigen-specific SED B cells, we find migration of cells towards the GC. Following antigen injection into ligated intestinal loops containing PPs, 40% of antigen-specific SED B cells bind antigen within 2 h, whereas unspecifc cells do not, indicating B cell-receptor involvment. Antigen-loading is not observed in M cell-deficient mice, but is unperturbed in mice depleted of classical dendritic cells (DC). Thus, we report a M cell-B cell antigen-specific transporting pathway in PP that is independent of DC. We propose that this antigen transporting pathway has a critical role in gut IgA responses, and should be taken into account when developing mucosal vaccines.

## Abstract (web summary)

Gut lumen antigens must be continuously sampled by the immune system to maintain proper immune homeostasis. Here the authors show that activated CCR6^+^CCR1^+^GL7^-^ gut B cells retrieve lumen antigens from specialized M cells and transfer them across the subepithelial dome in the Peyer’s patch to contribute to the maintenance of gut humoral immunity.

## Introduction

The Peyer′s patches (PP) in the small intestine is the site for the most intense activation of B cells in the whole body^1–3^. The site is exposed to a myriad of food and microbial-derived antigens, causing the PPs to host a large number of germinal centers (GC) at any given time point^4^. Therefore, it is reasonable to assume that the microenvironment and immune regulation at this site are quite different from that of secondary lymph nodes or the spleen^3^. The microanatomy of the PP includes B cell follicular areas, inter-follicular regions enriched for T cells and a follicle-associated epithelium (FAE), which acts as an interface between the lumen and the underlying sub-epithelial dome (SED)^3^. The specialized microfold (M) epithelial cells of the FAE can take up antigens derived from bacteria, viruses, fungi, toxins, inert particles and immune complexes^5–7^. A lack of M cell function reduces gut IgA responses dramatically^8,9^ and IgA coating of bacteria has been found to enhance the efficiency with which bacteria are captured and transcytosed to the SED^7,10–12^. The uptake mechanism is facilitated by expression of certain receptors, such as Dectin-1 and glycoprotein-2 (GP2), that  bind whole bacteria or soluble antigens^5–7,13,14^. However, it is unclear how antigen is further transported from the SED to the GC of the PP.

Adjacent to the M cells is the SED region that hosts multiple cell types, including different subsets of dendritic cells (DCs), macrophages, neutrophils, B and T cells^3,15,16^. At their basal side M cells form pockets that contain DCs, as well as T and B cells^17–21^. Whereas our knowledge about M cells and their function has grown in recent years our understanding of the complex environment of the SED is still incomplete. Although we know of the diversity of cell subsets in SED, the actual function of this site for IgA responses is still poorly understood^6,15,21,22^. A prerequisite for localization of B and T cells to the SED is their expression of CCR6 as the site produces the CCR6 ligand CCL20^1,22^. Elegant studies by Cyster and co-workers have identified activated IgD^+^ B cells that are  at a pre-GC stage in SED, where they express activation-induced cytidine deaminase (AICDA), and, hence, could undergo class-switch recombination (CSR) to IgA^1^. When activated B cells start to express CCR6 they can move into the SED, where they interact with other cells, in particular, CD11b^+^ CD8^−^ DCs^23,24^. These DCs express integrin αvβ8 that can activate latent transforming growth factor β (TGFβ), the prime switch factor required for IgA CSR^25–29^. Although the key source of latent TGFβ appears to be B cells themselves ^3^, also CD4 T cells, FDCs and DCs can produce the cytokine^4^. B cells proliferate in the SED, which is considered a prerequisite for CSR^16,30^. Hence, it is possible that most of the IgA CSR in SED depends on pre-GC B cell interactions with the DCs. Nevertheless, it is believed that the IgA-switched B cells can return to the follicle by directed migration and participate in the GC response^2^. Whether returning B cells constitute an important population in an ongoing immune response is largely unknown. We have identified that in CD40-deficient mice substantial IgA CSR against T cell-independent antigens occurs in the absence of GC in the PP^31^. Therefore, IgA-switched B cells in SED could primarily represent T cell-independent responses. Even though other studies have emphasized the CD40-dependency of the SED response^1^, most studies have indicated that the GC is the dominant site for IgA CSR in response to T cell-dependent antigens and that disruption of the GC in PP significantly reduces the number of IgA plasma cells in the mucosa^2,32^. Thus, it is debatable whether the presence of B cells in SED is primarily for IgA CSR or whether there may be other functions critically achieved at this site. For example, Mach et al. described a possible function of B cells in SED to maintain a mature phenotype of the M cell^33^. Furthermore it has been speculated that activated B cells (IgD^−^IgA^+^) could transport antigen acquired from M cells in the SED, but experimental evidence of this function is largely lacking^3,34,35^.

While B cells in SED express AICDA, critical for CSR, this factor is more strongly expressed in the GC, where follicular dendritic cells (FDC) form a network that organizes the GC reaction and functions as a deposit of antigen^1,2,36^. In lymph nodes and spleen the GC is an area where proliferating Ki67^+^ B cells express GL7, but this definition may not fully apply to the PP^32^. B cells in the GC express BCL6, a transcription factor critical for regulation of the expression of the *Gpr138* (*Ebi2*), *Cxcr4* and *S1Pr1* genes, which control positioning of the B cell in the follicle^37^. The GC is organized into a light (LZ) and a dark zone (DZ) and the former hosts the FDC network that carries antigen-complexes, critical for clonal selection and affinity maturation through somatic hyper mutation (SHM) of the IgA response^38–40^. In the DZ activated B cells undergo extensive cell divisions and this compartment hosts a network of CXCL12^+^ reticular cells (CRCs) that attract CXCR4^high^ B cells to migrate into this zone^38,39^.

Despite much progress in recent years we still lack a detailed understanding of how IgA induction is regulated in PP and, in particular, the specialized functions of the GC and SED^2–4,16^. We have developed a model system to study mucosal antigen-specific B cell responses based on GFP-labeled NP-specific B1–8^hi^ IgH knock-in B cells and oral immunization with the hapten (4-hydroxy-3-nitrophenyl acetyl; NP) conjugated to cholera toxin (CT)^16,32,41^.

Using this model, we here have explored the regulation of GC B cells in PP and compared this with systemic lymphoid tissues. In particular, we investigated the expression of GL7 and whether this expression correlates to a B cell function or a stage of differentiation. Most importantly we investigated the role of GL7-negative GFP^+^ B cells that express CCR6 and are in close contact with the M cells in the SED. We found that these NP-specific B cells bound antigen injected into a ligated loop of the small intestine, and then migrated from the SED to the GC. This M cell-B cell pathway was lost in M cell deficient mice, but was found intact in mice depleted of DC. We propose that this pathway plays an important role in maintaining the GC response in the PP and subsequently also for gut IgA responses.

## Results

### Most antigen-specific GC B cells in PP are GL7-negative

**Fig. 1:**
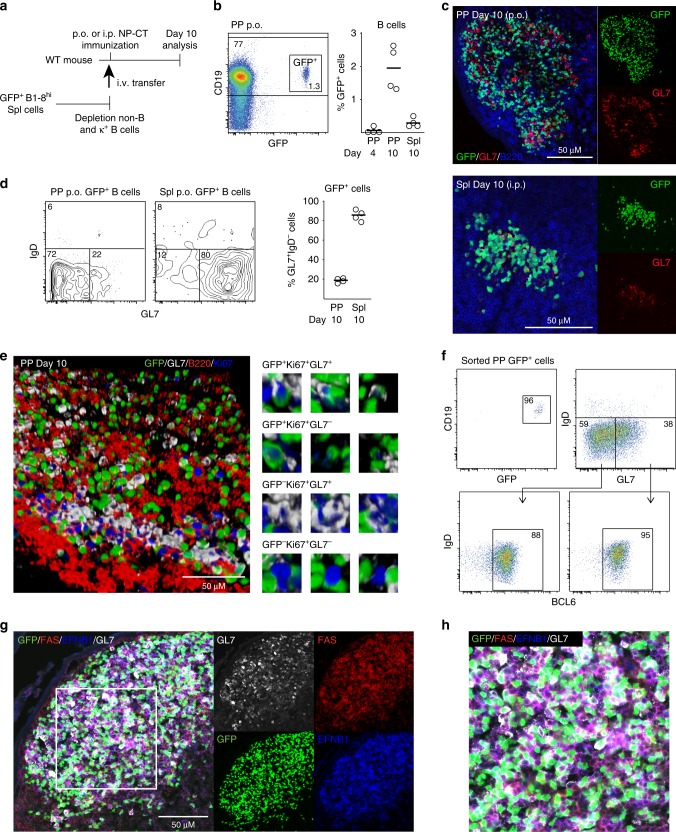
A majority of Peyer′s patch (PP) germinal center (GC) B cells lack expression of GL7. **a** A schematic depiction of the experimental model used to study specific B cell responses in the PP and spleen following per oral (p.o.) or intraperitoneal (i.p.) immunization. **b** Gating strategy and percentage of NP-specific GFP^+^ B cells of all CD19^+^ B cells in PP on day four and ten or in the spleen (Spl) on day ten after a single NP-CT p.o. or i.p. immunization. **c** Representative microscopy images of PP and spleen tissue sections showing GL7^+^ (red) and GL7^−^ GFP^+^ (green) B220^+^ (blue) B cells in PP GC following immunization. **d** Flow cytometry contour graphs and percentage  of GL7^+^ activated IgD^−^ GFP^+^ B cells in PP and in the spleen on day ten following p.o. and i.p. immunizations, respectively. **e** 3D confocal microscopy image of a GC in PP with GL7^+^ (white) or GL7^−^ proliferating Ki67^+^ (blue) GFP^+^ (green) B cells co-labeled for B220^+^ (red). Close-ups of different labeling patterns of activated NP-specific GFP^+^ or non-specific endogenous GFP^-^ B cells in the GC image to the right. **f** A representative intracellular flow cytometry analysis of sorted GFP^+^ B cells of pooled PPs from two mice on day ten following oral immunization with NP-CT to assess the frequency of BCL6-expressing activated IgD^−^ GL7^+^ or GL7^−^ NP-specific B cells. **g**, **h** PP sections were labeled with FAS (red), EFNB1 (blue) and GL7 (white) to better show the demarcation of the GC reaction. Activated GFP^+^ B cells (green) are mostly within the GC borders while variably expressing GL7 (white). These are results from at least three independent experiments with 3–5 mice in each. Source data are provided as a Source Data file

Since PP constantly host GCs, it has been nearly impossible to study antigen-specific B cell responses using traditional mouse models and immunization approaches. To overcome this limitation, we have developed an adoptive transfer model based on NP-specific B1–8^hi^ IgH knock-in λ-expressing GFP^+^ splenic B cells and NP-hapten conjugated to cholera toxin (NP-CT) as an oral immunogen to study gut IgA responses^16,32,41^ (Fig. 1a, b). GL7 is a B cell activation marker that is upregulated before and during a GC response^42^. Following an oral immunization with NP-CT we found that a majority of NP-specific GFP^+^ B cells in the PPs were found in the GC compartment (Fig. 1c). Surprisingly, whereas most of the GFP^+^ B cells were IgD^−^ (>90%), only 20–25% of these cells expressed GL7 (Fig. 1d). This phenotype was specific for PP as following an i.p. immunization with NP-CT we identified that 80% of the activated IgD^−^ GFP^+^ B cells in the spleen were GL7^+^ and found in classical GC, suggesting that the regulatory microenvironments may differ between the two sites (Fig. 1c, d). Of note, neither the route or number of immunizations, the source of adoptively transferred naïve NP-specific B cells, splenic or isolated from the PP, or the time after immunization changed the uniquely low frequency of GL7-expressing activated B cells in the PP following oral immunization (Supplementary Fig. 1a–e).

**Fig. 2:**
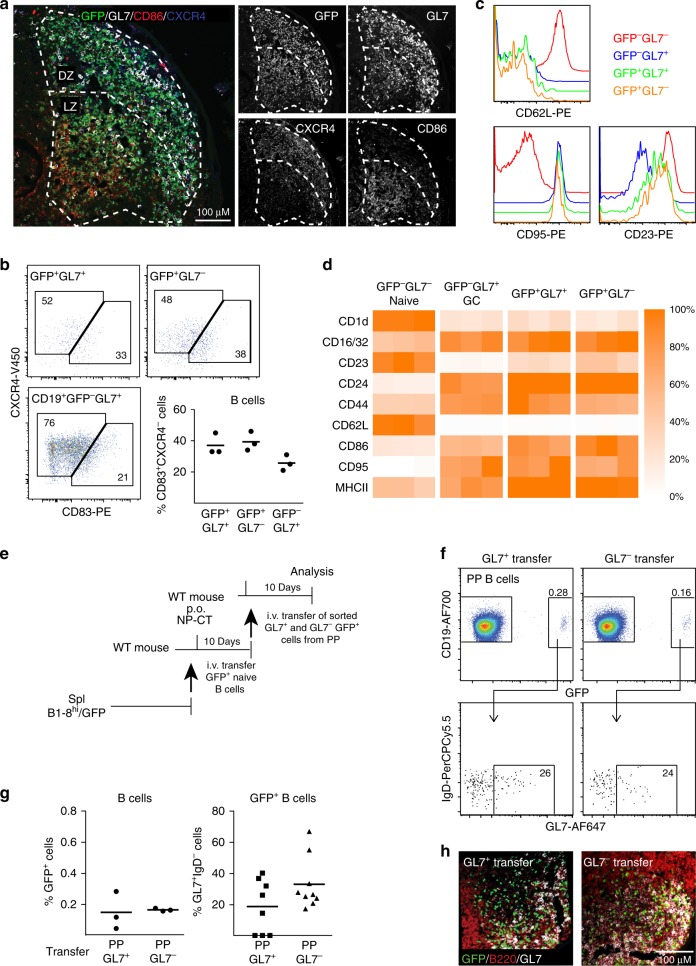
Location and activation status of GL7^+^ and GL7^−^ antigen-specific B cells in Peyer’s patch (PP) germinal centers (GC). **a** Representative microscopy image showing that NP-specific GFP^+^ B cells (green) localize both to the light, CD86^+^ (red), and dark, CXCR4^+^ (blue), zones of the GC (GL7^+^) in PPs following p.o. immunization with NP-CT. **b** Flow cytometry dot-plots demonstrating expression of light (CD83^+^) and dark (CXCR4^+^) zone phenotypic markers in GL7^+^ and GL7^−^ GFP^+^ and endogenous GL7^+^ GFP^−^ B cells using the gating scheme in Supplementary Fig. 2a. **c**, **d** Flow cytometry histograms and heat maps depicting expression of surface markers on GL7^+^ or GL7^−^ NP-specific GFP^+^ or endogenous GFP^−^ B cells in PP following a single p.o. immunization. **c** Representative flow data demonstrating the expression of CD62L, CD95 and CD23. **d** Heatmaps depicting the relative expression (MFI[sample]/MFI[maximum for any sample]) for the indicated markers in three mice. **e** Model used for adoptive transfer of sorted GL7^+^ or GL7^−^ activated GFP^+^ B cells from PP to a “naïve” host that had been given NP-CT 24 h prior to cell transfer. **f**, **g** At 8–10 days following the transfer of sorted activated GFP^+^ B cells, the frequency of activated GFP^+^ B cells of all CD19^+^ B cells in PPs of the recipient mice and their distribution in GL7^+^ and GL7^−^ cell populations were determined using flow cytometry. **h** A representative image demonstrating the GC location of sorted and transferred GL7^+^ or GL7^−^ GFP^+^ (green) B cells in the PP of recipient mice. Sections were stained with B220 (red) and GL7 (white) antibodies to reveal the position of the GC. These are representative data from at least three independent experiments with 3–5 mice in each experiment giving similar results. Source data are provided as a Source Data file

An extended analysis of frozen tissue sections revealed that in the PP GC areas proliferating Ki67^+^GFP^+^ B cells were both GL7^+^ and GL7^−^ (Fig. 1e). Importantly, we also observed that endogenous GFP^−^ Ki67^+^ GC PP B cells could be GL7^−^ and, hence, this phenotype was not unique to our transferred NP-specific B cells (Fig. 1e). A vast majority of GL7^−^ (88%) as well as the GL7^+^ (95%) NP-specific GFP^+^ B cells expressed BCL6, indicating that both populations were GC B cells, which was further confirmed by their expression of EFNB1 and FAS (Fig. 1f–h). Indeed, a detailed analysis of BCL6 expression in GL7^+^ and GL7^−^ activated GFP^+^ B cells from PP and spleen by flow cytometry revealed similar BCL6 expression levels, which was further supported by RNAseq data (Supplementary Fig. 1f–i). The GL7^−^ and GL7^+^ GFP^+^ B cells were distributed to both the light and dark zones of the GC and shared expression of zone specific markers, indicating that they do not associate with the functional features of these respective areas (Fig. 2a, b). Moreover, an extended phenotypic analysis of the GFP^+^IgD^−^CD138^−^ PP B cells by flow cytometry revealed that the two populations were identical with regard to a panel of activation and differentiation markers and highly similar to the endogenous GL7^+^GFP^−^ GC B cell population, but phenotypically distinct from naïve GL7^−^IgD^+^GFP^−^ B cells (Fig. 2c, d, Supplementary Fig. 2a).

To investigate the stability of the GL7^−^ phenotype we sorted highly enriched GL7^−^ and GL7^+^ GFP^+^ B cells (>90% purity) from PP of immunized mice and transferred these into naïve hosts that had been given NP-CT orally 24 h prior to transfer (Fig. 2e; Supplementary Fig. 2b). Roughly 25% of the GFP^+^ B cells expressed GL7 after this secondary transfer of activated GFP^+^ B cells irrespective of if the transferred B cells were GL7^+^ or GL7^−^ (Fig. 2f, g). This suggested that the GL7-phenotype was reversible and that expression of GL7 in PP did not associate with a distinct stage of differentation or function. Both populations also clearly located to the GC of PP in the recipient mouse after transfer, consistent with our earlier observation of re-utilization of pre-existing GC in multiple PP by orally activated PP B cells following adoptive transfer^41^ (Fig. 2h).

### Gene expression profiles in GL7^−^ and GL7^+^ PP B cells

**Fig. 3:**
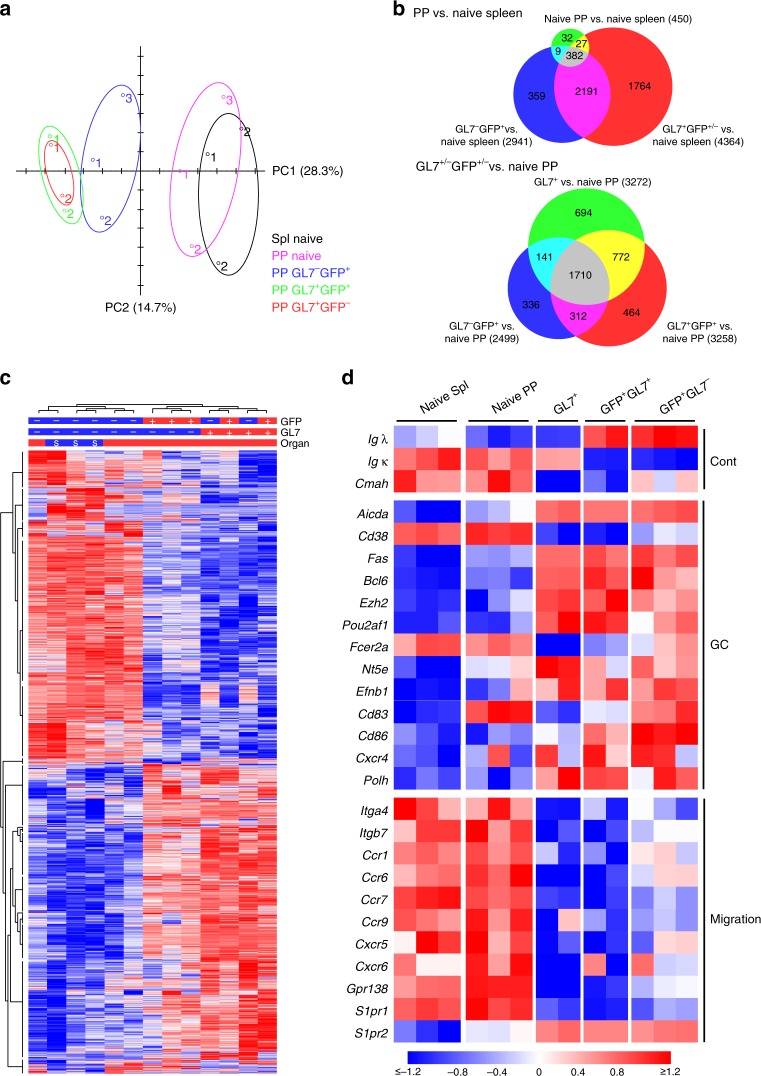
RNASeq analysis of GL7^+^ and GL7^−^ antigen-specific Peyer’s patch (PP) B cells. **a** GFP^+^ antigen-specific B cells were sorted into GL7^+^ or GL7^−^ populations on day ten following an oral immunization with NP-CT and their gene expression profiles were analyzed using RNAseq. Concomitantly GL7^−^ (naïve) and GL7^+^ B cells from PP and GL7^−^ B cells from the spleen of unimmunized control mice were sorted for a comparative analysis of gene expression profiles. A principle component analysis of RNAseq data demonstrated global gene expression similarities between GL7^+^ and GL7^−^ GFP^+^ B cells that were distinctly different from GFP^−^ naïve splenic or PP B cells, but similar to endogenous activated GFP^−^ GL7^+^ GC B cells. **b** Venn diagrams comparing gene expression changes between groups. In the upper panel, naïve GL7^−^ (*n* = 3 samples), activated PP GFP^+^GL7^−^ (*n* = 3) and activated GFP^+/−^GL7^+^ PP (*n* = 4) PP B cells were compared to GL7^−^ naïve splenic B cells (*n* = 3), and in the lower panel activated PP GFP^+^GL7^−^ (*n* = 3), GFP^+^GL7^+^ PP (*n* = 2) and GFP^−^GL7^+^ PP (*n* = 2) were compared to naïve GL7^−^ PP B cells (*n* = 3). Genes with a fold change > 1.5 and FDR adjusted *p*-value < 0.05 were counted, and the figures in each Venn field indicate the number of shared genes between groups. **c** Heat map of global gene expression in the PP B cell subpopulations compared to that of sorted naïve splenic B cells. **d** Heat map depicting the mRNA expression of selected genes in different B cell subpopulations, demonstrating differences between GL7^+^ and GL7^−^ NP-specific GFP^+^ B cells with regard to gene expression signatures associated with migratory cues but similarities in expression of GC genes. Differences in gene expression are indicated the same in **c** and **d** with the scale at the bottom being applicable to both

To get a better understanding of the relationship between the GL7^+^ and GL7^−^ PP B cells we performed an RNASeq analysis of sorted GFP^+^ B cells (Supplementary Fig. 3). The gene expression patterns exhibited by these two subsets were compared to those of endogenous GL7^+^ and GL7^−^ (naïve) GFP^−^ PP B cells and splenic GL7^−^ (naïve) B cells from unimmunized mice. Principal component analysis (PCA) of global gene expression corroborated our previous result, demonstrating that activated GL7^−^ GFP^+^ PP B cells were similar, albeit not identical, to GL7^+^ GFP^+^ PP B cells (Fig. 3a). The majority of upregulated or downregulated genes, as compared to GL7^−^ naïve splenic B cells, were shared between GL7^+^ and GL7^−^ activated GFP^+^ PP B cells (Fig. 3b, upper panel). Few genes differed in expression between unimmunized naïve PP and splenic B cells; these likely represented some contaminating activated B cells in the naïve PP B cell population. However, global gene expression analysis demonstrated that the activated GL7^−^ GFP^+^ PP B cells were distinctly different from naïve PP B cells (Fig. 3b, lower panel). Clustering of individual samples based on gene expression showed that GL7^−^ and GL7^+^ GFP^+^ B cells formed distinct subgroups, which clearly separated from naïve B cells (Fig. 3c). A total of 535 genes were identified as differentially expressed genes between GL7^+^ and GL7^−^ PP B cells (FDR *p*-value < 0.05). Based on a gene ontology (GO) analysis, these were highly enriched for genes encoding molecules involved in antigen and immunoglobulin receptor binding, immune effector functions, cell migration or tissue localization (Supplementary Data 1). For example, in addition to the gene encoding CMAH, the enzyme responsible for reducing GL7-expression on resting B cells^43^, there were several receptor encoding genes involved in homing or tissue localization of activated B cells, including *Ccr6*, *Gpr183* (*Ebi2*), and *Itgb7*, that were significantly differentially expressed (Supplementary Data 2).

As expected, GFP^+^ PP B cells were clearly enriched for cells expressing λ-light chains. CD23 gene-expression was also higher in both populations of GFP^+^ B cells than in endogenous GL7^+^ B cells, in agreement with our earlier phenotypic analysis (Fig. 3d). Importantly, GL7^−^ GFP^+^ B cells expressed similar levels of the *Bcl6* gene as the GL7^+^GFP^+^ PP B cells, confirming their GC phenotype (Fig. 3d and Supplementary Fig. 1g). Moreover, both GL7^+^ and GL7^−^ GFP^+^ PP B cells expressed increased levels of *Aicda* (*Aid)*, *Fas* (*Cd95*), *Ezh2*, *Pou2af2* (*Oca-B*), and *Nt5e* (*Cd73*) mRNA transcripts as compared to those seen with naïve B cells (Fig. 3d). The expression of the *Cd38* gene was downregulated, and *Cd86* and *Cxcr4* genes were upregulated in both populations, supporting that the GL7^−^ and GL7^+^ GFP^+^ B cell populations localized to both the light and dark zones (Fig. 3d). Interestingly, the *Cd83* gene expression was higher in GL7^−^ PP B cells and comparable to that in naïve PP B cells (Fig. 3d). More importantly, though, mRNA encoding genes associated with B cell movement and homing, including integrin β7 (*Itgb7*), the chemokine receptors *Ccr1*, *Ccr6*, *Cxcr5*, and *Cxcr6*, and the oxysterol receptor *Gpr183* (*Ebi2*), were more highly expressed in GL7^−^ than in GL7^+^ GFP^+^ PP B cells (Fig. 3d). The higher expression of *Ccr6* and *Ccr1* mRNA could indicate trafficking of GL7^−^ GFP^+^ B cells to the subepithelial dome (SED) or follicle associated epithelium (FAE)^1,44^, but expression of the CCR6 gene has also been associated with a pre-memory stage of B cell differentiation^45–47^.

### GL7^−^ GFP^+^ PP B cells distribute to both the GC and SED

**Fig. 4:**
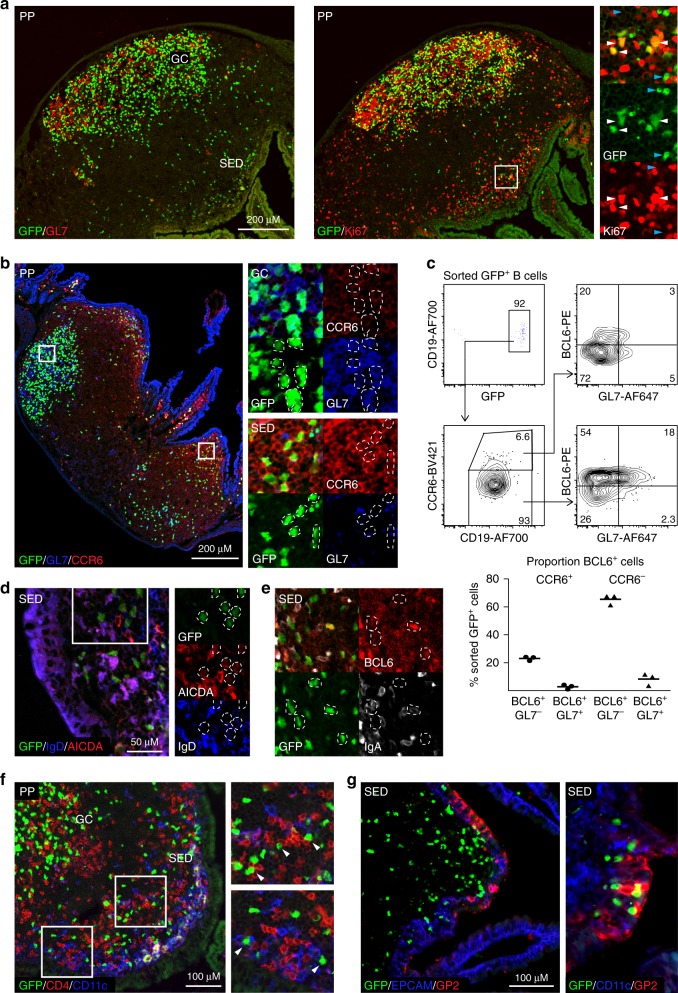
Antigen-specific activated GL7^−^ B cells are present in the Peyer’s patch (PP) sub-epithelial dome (SED) during an ongoing response. **a**, **b** Representative microscopy images showing activated and proliferating B cells in PP and SED. **a** The right panel and close-ups show GFP^+^ (green) proliferating (Ki67; red) and resting B cells that do not express GL7 in SED, with proliferating GL7^+^ (red; left panel) or GL7^−^ B cells in the GC ten days after an oral immunization with NP-CT. **b** Activated GFP^+^ (green) B cells in SED are GL7^−^ (blue) and express CCR6 (red), while GC GFP^+^ B cells do not express CCR6 regardless of GL7 expression. **c** A representative intracellular flow cytometry analysis of sorted GFP^+^ B cells from pooled PP from two mice on day ten following an oral immunization with NP-CT to quantitively assess BCL6 and GL7 expression on CCR6^-^ (GC) and CCR6^+^ (SED) NP-specific B cells. A majority (>70%) of GFP^+^ CCR6^−^ B cells in GC expressed BCL6 and of those one quarter also expressed GL7, whereas only around 20% of the CCR6^+^ SED B cells were BCL6^+^ of which essentially all lacked GL7. **d**, **e** Confocal microscopy images of the PP SED region following oral immunization with NP-CT showing expression of **d** AICDA (red) and IgD (blue) and **e** IgA (white) and BCL6 (red) in GFP^+^ (green) B cells. **f** Confocal microscopy demonstrating close contact between NP-specific GFP^+^ (green) B cells and CD4 T cells (red) and CD11c cells (blue) (white arrowheads in the close-ups) and **g** close interactions between NP-specific GFP^+^ B cells (green) and GP2^+^ (red) M cells in the EPCAM^+^ epithelial layer (blue) of the follicle associated epithelium of the PP. These are representative data from at least 3 independent experiments with each experiment giving similar results. Source data are provided as a Source Data file

Whereas flow cytometry only quantified the relative presence of the GFP^+^ B cell populations, their actual location in the PP was unclear and, therefore, we performed confocal microscopy of tissue sections. We analyzed frozen PP tissue sections on day 10 after oral immunization and found proliferating Ki67^+^ GFP^+^ B cells in both the GC and SED regions (Fig. 4a). Significant numbers of CCR6^+^ GFP^+^ B cells expressing no or low levels of GL7 were observed in SED, which contrasted with GFP^+^ B cells in the GC, which were CCR6^low^, regardless of GL7-expression (Fig. 4b). The proportion of cells that expressed BCL6 among CCR6^+^ GFP^+^ B cells in SED was lower than that found within CCR6^−^ GFP^+^ B cells in the GC (Fig. 4c). Noteworthy, GFP^+^ B cells in SED were IgD^−^ and had low, but detectable, expression of AICDA (Fig. 4d). Some of the GFP^+^ B cells in SED had switched to IgA, but no sign of plasmablast differentiation was observed as IgA expression levels were low (Fig. 4e). Rather, these activated B cells expressed EFNB1 and FAS (CD95), albeit at low levels, suggesting that they may have emanated from the GC (Supplementary Fig. 4a). Alternatively, this observation is consistent with the notion that IgA CSR may occur at a stage of B cell differentiation prior to or even independent of GC formation^1,41^^,^. Close interactions of activated GFP^+^ B cells with both DCs and CD4^+^ T cells were also observed in the SED (Fig. 4f). However, most strikingly, many GFP^+^ B cells were intricately juxtaposed to the epithelium centrally in SED, seemingly in close contact with the epithelial layer (Fig. 4g). This interaction was primarily restricted to GP2-expressing M cells, rather than EPCAM^+^ epithelial cells in general, with close contacts between M cells and IgA^+^ and IgA^−^ GFP^+^ B cells (Fig. 4g and Supplementary Fig. 4b). This suggested that a prominent function of the GL7^−^ GFP^+^ B cells in SED could be to acquire luminal antigen from the M cells. Interestingly, blocking of α4β7 with a specific mAb in vivo at day eight after an oral immunization with NP-CT did not reduce the number of GFP^+^ B cells in PP, indicating that during the response recruitment of B cells from the circulation was not required to maintain the PP response (Supplementary Fig. 4c)^48^. Thus, taken together our findings indicated that during an ongoing B cell response in the PP, activated IgD^−^ NP-specific B cells in SED closely interact with M cells, suggesting an antigen-sampling function, and, perhaps, the migration of these B cells from SED to the GC with captured antigen^9^.

### Migration of antigen-specific B cells from SED to the GC

**Fig. 5:**
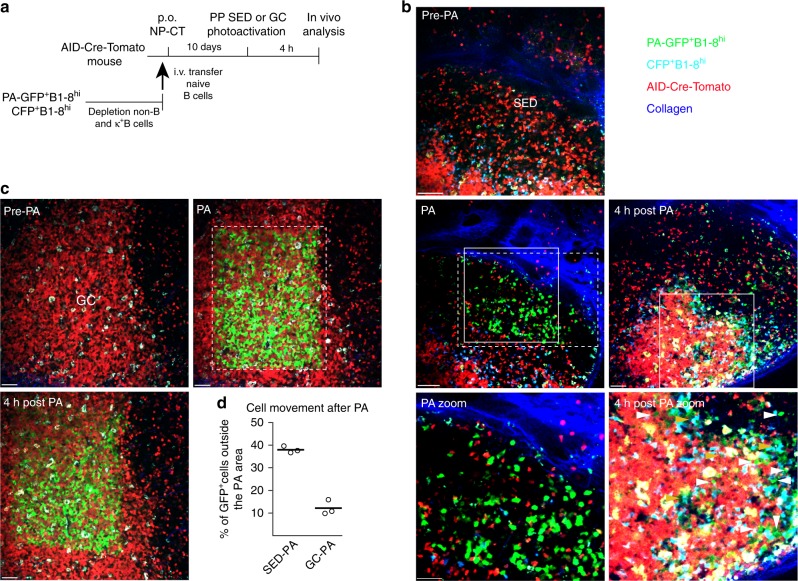
Migration of activated NP-specific B cells from the SED to GC regions of the Peyer’s patch following oral immunization. **a** Schematic representation of the model used to detect movements of B cells in the PP by photoactivation. B1–8^hi^ B cells expressing PA-GFP or CFP were adoptively transferred into AID^Cre/+^ Rosa26^fl-stop-fltdTomato^ mice that were subsequently orally immunized with NP-CT. At 10 days after transfer, PPs were exposed and SED or GC areas were photoactivated based on CFP and tdTomato expression and the position of the photoactivated cells was subsequently analyzed 4 h later. **b** Photoactivation was guided to the SED region based on the presence of CFP^+^ cells. Four hours after activation approximately 40% of the GFP^+^ B cells had left the photo-converted area in SED. These were often found close to or within the GC at this point (marked with white arrowheads). In addition, macrophages were evident by auto-fluorescence (marked with orange arrowheads; also see Supplementary Fig. 5a). **c** The GC was detected based on the large density of tdTomato^+^ cells in the area. Four hours after photoactivation most of the PA-GFP^+^ B cells remained within the GC area. **d** The percentage of B cells that had migrated from the photoactivated area within 4 h. The analysis represents one experiment performed on three distinct PP. Photoactivated areas are marked with dashed line rectangles and close up areas with normal line quadrants. Similar results were obtained in 3 independent experiments. Scale bar = 80 µm. Source data are provided as a Source Data file

The presence of antigen-specific GFP^+^ B cells in both the SED and GC compartments suggested that B cells may be able to migrate between the two regions. Indeed, some antigen specific GFP^+^ B cells were consistently observed between the GC and SED in tissue sections of PP (Fig. 4a, b and Supplementary Fig. 4a). To directly address this question, we used photoactivtable (PA-GFP) B1–8^hi^ B cells and in situ-labled B cells in living mice to track their migration 8 days following an oral NP-CT immunization^49,50^. To mark the site for photoactivation, we co-transferred B1–8^hi^ B cells expressing cyan fluorescent protein (CFP) to AID^Cre/+^ Rosa26^tdTomato/+^ mice that served as landmarks for the SED and GC, respectively (Fig. 5a). Tracking of B cells photoactivated in the SED region showed that nearly 40% of the activated PA-GFP^+^ B cells had left the SED after 4 h (Fig. 5b–d). Many of these B cells were found close to or whithin GCs at this time point (Fig. 5b and Supplementary Fig. 5a, b). In contrast, when B cells in PP GC were photoactivated, only 10% of the PA-GFP cells had left the photoactivation area within the same time-frame (Fig. 5c, d). B cells that had left the GC PA area were still within the GC, but cells that left the SED PA area were almost exclusively detected in or in proximity of the GC (Supplementary Fig. 5b). Thus, we detected rapid migration from SED to GC area but the data failed to support the notion of a migration from GC to SED, possibly due to less frequent events.

### NP-specific CCR6^+^GL7^−^ B cells sample luminal antigen in SED

**Fig. 6:**
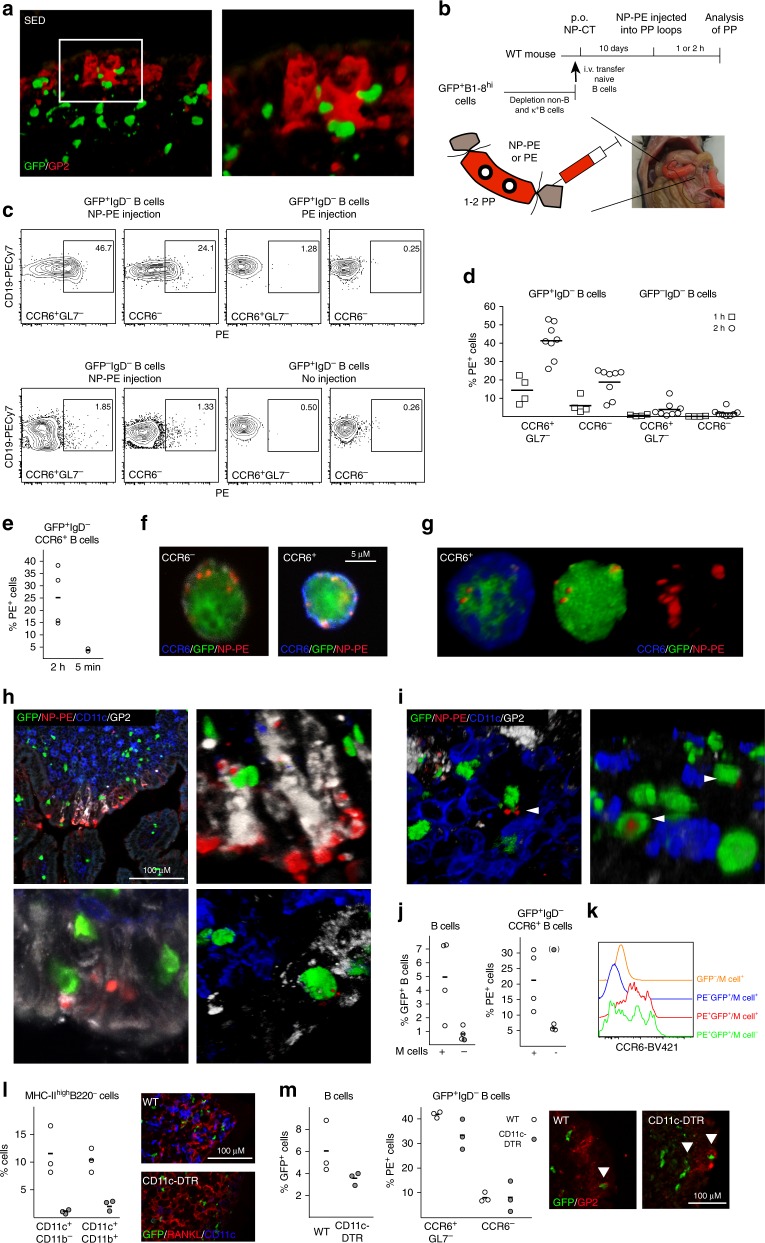
Peyer’s patch M cells provide antigen-specific B cells with luminal antigen for transport towards the germinal center area. **a** A 3D confocal microscopy image of GFP^+^ B cells (green) in contact with GP2^+^ M cells after oral immunization with NP-CT. **b** Schematic representation of a ligated loop model used to study antigen up-take from the lumen. **c** Flow cytometry contour plots analyzing antigen-specific binding of NP-PE to GFP^+^ and endogenous GFP^−^ B cells. The left panels show NP-PE binding to antigen-specific (top) or endogenous (bottom) B cells 2 h after NP-PE injection, and the right control experiments with loops injected with unconjugated PE or PBS. **d**, **e** The graphs show the binding of NP-PE to SED (CCR6^+^GL7^−^), non-SED (CCR6^−^) GFP^+^ or endogenous GFP^-^ B cells 5 min, 1 or 2 h after injection. **f**, **g** Sorted CCR6^+^ (blue) and CCR6^-^ PE^+^ B cells were fixed and spread on microscope slides to determine the cellular distribution of NP-PE (red). **h** Representative confocal microscopy images show antigen accumulation (NP-PE; red) on M cells (GP2^+^; white) 1 h after NP-PE inoculation with GFP^+^ B cells (green) accessing antigen in and/or below M cells. **i** Antigen-specific B cells (GFP^+^; green) carrying antigen (NP-PE; red) while not contacting M cells (GP2^+^; white) or DC (CD11c^+^; blue). **j** The proportion GFP^+^ B cells in PP following oral immunization and the frequency of NP-PE binding SED B cells two hours after NP-PE injection into ligated loops of M cell deficient RANK-FL/Villin-Cre^+^ or control mice are shown. Averages were computed without an outlayer (gray) based on less than 30 GFP^+^ events. **k** A histogram depicting CCR6 expression in NP-PE^+^ and control B cells from M cell deficient or control mice. **l** DC were depleted from CD11c-DTR transgenic mice 24 h after a DT injection, with no CD11c-expressing cells in the RANKL-expressing (red) SED. **m** The left diagram show the proportion GFP^+^ B cells after DT-mediated DC depletion 24 h before loop experiments  and in control mice, the middle graph the frequency of NP-PE binding B cells in ligated loops, and the microscopy photo to the right interactions between antigen-specific B cells (green) and M cells (GP2^+^; red). Source data are provided as a Source Data file

We observed close interactions between antigen-specific B cells and M cells in the SED, suggesting a potential antigen-sampling function (Fig. 6a). To determine if this was the case, we analyzed the ability of CCR6^+^ GFP^+^ B cells within the SED to take up luminal antigen. Because we failed to detect fluorescent protein in PPs after oral gavage we directly delivered fluorescent NP-PE into ligated loops of the small intestine in mice that had been orally immunized with NP-CT 10 days earlier (Fig. 6b). We examined single cell suspensions of PP B cells for bound antigen at 1 h following inoculation using flow cytometry and found that GFP^+^, but not endogenous GFP^−^, B cells carried NP-PE (Fig. 6c, d, Supplementary Fig. 6a). After 2 h, 40% of CCR6^+^ GL7^−^ SED NP-specific GFP^+^ B cells were PE^+^, while 20% of CCR6^−^ GFP^+^ B cells also had bound PE-antigen at this point (Fig. 6c, d). By contrast, endogenous GFP^-^ B cells failed to bind NP-PE (Fig. 6c, d). Essentially no NP-PE binding could be observed on GFP^+^ B cells when samples were taken 5 min after NP-PE installation, demonstrating that the NP-PE binding was not due to leakage or rapid tissue diffusion (Fig. 6e). GFP^+^ B cells isolated from PE-only injected loops or from PPs outside of the ligated loops had no bound NP-PE, confirming that the observed binding was antigen-dependent and B cell specific (Fig. 6c). Importantly, the frequency of PE^+^ B cells was substantially higher among CCR6^+^ GL7^−^ SED B cells than in CCR6^−^ GL7^+/−^ GC B cells at both 1 and 2 h, supporting the notion of a migration of GFP^+^ B cells from the SED to the GC (Fig. 6c, d). Sorting of NP-PE binding CCR6^+^ and CCR6^−^ B cells revealed that the antigen was localized in clusters at the cell surface (Fig. 6f, g and Supplementary Fig. 6b). In tissue sections of PP from ligated loops injected with NP-PE, the antigen was enriched in M cell dense regions of the epithelium (Fig. 6h and Supplementary Fig. 6c, d). Antigen was also present at the basal area of the M cells, i.e. in the pockets to which B cells were located. Deeper in the tissue GFP^+^ B cells carried antigen as complexes at the cell surface in a similar manner as was observed in the sorted CCR6^+^ GFP^+^ B cells (Fig. 6i and Supplementary Fig. 6e).

To determine the importance of M cells for transfer of luminal antigen to the GFP^+^ B cells in the SED we performed a ligated loop experiment in M cell deficient mice^51^. These mice are specifically deficient in *Tnfrsf11a* (which encodes RANK) in *Vil1*-expressing intestinal epithelial cells^9,52^ and subsequently lacked GP2-expressing M cells in the gut (Supplementary Fig. 6f). As expected from the previously described role of M cells in taking up and transporting antigen to the SED region in the PP^9^, the number of GFP^+^ B cells was lower in PP after oral NP-CT immunization in these mice (Fig. 6j). However, of the GFP^+^ CCR6^+^ B cells present in the SED region of M cell deficient mice only few carried NP-PE (5%) after injection of NP-PE into the ligated loops, demonstrating a critical role for the M-cell for antigen uptake and delivery to the GFP^+^ B cells in the SED (Fig. 6j). Interestingly, most antigen-binding B cells found in M cell deficient mice were not CCR6^high^ GFP^+^ B cells, as found in wild-type mice and, hence, not the B cell phenotype previously described to interact with M cells (Fig. 6k)^22^. Thus, M cells were required for GFP^+^ PP B cells to access and bind luminal NP-PE antigen in the SED during an ongoing immune response following oral immunization.

Because previous studies, as well as our own study, identified that DCs in the SED can be in close contact with the M cells and can capture luminal antigen, either directly by transepithelial transcytosis of dendrites or by taking up antigen delivered by M cells, we undertook experiments in a mouse model depleted of DCs^3,53^. We used the CD11c-DTR-model and performed the ligated loop experiment in mice injected with diptheria toxin (DT) at 9 days following an oral immunization with NP-CT^54^. We achieved an almost complete elimination of classical DCs (MHC class II^high^, CD11c^+^, CD11b^+/−^) in these mice, including the PPs, leaving essentially no CD11c^+^ cells in the SED region at day 10 when the experiment was done (Fig. 6l and Supplementary Fig. 6g). Despite this lack of DCs in the SED we observed only minimal reduction in the M cell dependent binding of NP-PE to GFP^+^ B cells in the SED (Fig. 6m). CCR6^+^ GFP^+^ B cells from SED in DC-depeleted mice had bound NP-PE to a similar extent as cells from WT mice and were in close contact with the M cells (Fig. 6m). Thus, we had found evidence for an M cell-B cell specific pathway, independent of DCs, for sampling and transportation of antigen from the SED to the GC in the PP. This M cell-B cell axis relied on activated GL7^−^ CCR6^+^ SED B cells, which used the specific B cell receptor for antigen binding.

## Discussion

In order to develop a strong IgA response to T cell dependent antigens in the gut the GC reaction in the PP has to be maintained for some time. However, we are largely lacking information on how antigens are delivered from the FAE to the GC of the PP. A particular problem to understand is how antigens can be selected at this site when there are myriads of antigens present in the lumen and arguably only minute amounts of an orally delivered antigen can be expected in the lumen. In this regard B cells with their receptors can function as antigen-specific binders making it possible to selectively support and sustain a GC reaction in a given PP over time. Because PPs consistently maintain GC in conventionally reared mice it has proven virtually impossible to examine antigen delivery and activation of antigen-specific B cells at this site. To bypass this limitation we have established a model system that allows us to follow antigen-specific B cell responses and to undertake detailed investigations of the immune regulation at the PP-inductive site^41^. In the present study we found that activated GFP^+^ B cells in the GC of PP were mostly GL7-negative and that during an ongoing immune response such cells were also observed in the SED, where they made close contact with the M cells of the FAE enabling binding of antigen. Antigen delivery experiments using ligated loops demonstrated that activated GFP^+^ B cells in the SED bind luminal antigen and photoactivation experiments showed that these GFP^+^ B cells could migrate towards the GC. Loop experiments in M cell deficient mice failed to show binding of antigen to GFP^+^ B cells in SED, while DC-depleted mice demonstrated unperturbed binding ability. Thus, during an ongoing response in PPs antigen can bind specific B cells in SED after M cell sampling of luminal antigen. In this way the ongoing GC reaction can selectively import luminal antigen, due to the specific BCR-mediated function of the activated CCR6^+^GL7^−^ B cells in the SED. Accordingly, only NP-specific B cells participated in the antigen transport, while GFP^−^ non-specific B cells were not involved. We believe this observation adds support to our hypothesis of how activated B cells in multiple PPs can re-utilize already existing GC for continued expansion and differentiation^45^. The re-utilization of GCs leads to strong clonal selection and a synchronization of the gut IgA response to include only high quality IgA antibodies. GCs in multiple PPs can this way secure presence of antigen by the M cell-B cell pathway and when antigen is lost in the gut lumen the GC reaction for a given antigen in the PP subsides.

Previous publications have reported on several possible functions of B cells in the SED region of the PP. For example, Mach et al. described close interactions between M cells and B cells as a mechanism to maintain the mature M cell phenotype^33^. These authors described in detail the high level of M cells with interactions with B cells, close to 80%, and with a ratio of 1:1 with a competitive edge of newly recruited B cells for M cell occupancy in the PP. Contrary to our study these investigators did not analyze B cell specific responses in PP, and were, thus, unaware of the strong preference of activated B cells to locate to the SED and close to the M cells in the FAE. Interestingly, we observed that the CCR6^+^ GFP^+^ B cells in SED also expressed increased levels of the *Ccr1* gene, which encodes the receptor for CCL9 that is uniquely produced by M cells in the PP and has been found critical for DC recruitment to the M cell^44^. Hence, this may explain the strong interaction between the B cells and the M cells in the SED. CCR6^+^ B cells have also been found critical for M cell differentiation and now the antigen-transporting function will be added to the list of functions that link the PP B cells and M cells in the SED^22^. The fact that the M cell-B cell axis was unperturbed in CD11c-DTR mice depleted of DC in SED, strongly argues for that DCs are dispensible for the antigen-transporting pathway that we have described. However, our study does not exclude that DCs still may be important for M cell functions as we only acutely depleted these cells after oral immunization and prior to the loop test. In fact, several publications attest to a critical interplay between DC and M cells in the SED^44^.

Another well documented event in the life of SED B cells is IgA CSR, which was detected by switch-circle transcripts, indicative of an ongoing CSR process. Since it was reported that CCR6-expression was CD40-dependent in SED B cells, the authors suggested that T cell-dependent rather than T cell-independent responses underwent IgA CSR in SED^1^. However, recently Lin et al. reported decreased IgA responses in CCR6-deficient mice associated with reduced Th17 and Tfh frequencies in the GC of PP^55^. Thus, IgA CSR was impaired also in the GC as a consequence of the CCR6-deficiency^55^. We have previously reported T cell-independent IgA CSR in PP of CD40-deficient mice at a stage (GL7^int^) prior to manifest GC. This IgA CSR could have occurred in SED, since AID expressing B cells appear to localize close to the epithelium in αβTCR-deficient mice^31,56^. In the present study we follow a T cell-dependent response and observe the presence of AID-expressing IgA^+^ CCR6^+^ GFP^+^ B cells in SED, suggesting that they could have undergone IgA CSR at this site. But, at present, we cannot exclude the possibility that GFP^+^ IgA-switched B cells had migrated from the GC to the SED. Although we failed to demonstrate a migration of B cells from the GC to SED using photoactivation we cannot exclude that the GC was formed first and then activated B cells migrated to the SED. Notably, we have here and in other publications documented extensive proliferation in SED, suggesting that even if few cells leave the GC and migrate to SED, they may expand there before returning^16^. It should be emphasized that photoactivation of B cells in gut tissues of living mice is very challenging because of the peristalsis of the intestine and the low percentage and slow pace of activated B cells leaving the GC. However, *Efnb1* and *Fas* (*Cd95*) expression in GFP^+^ SED B cells genes may support that they emanated from the GC^45^ and single cell immunoglobulin heavy chain sequencing have also suggested an active exchange between GC and SED^16^. Nevertheless, further studies are required to resolve how the GFP^+^ B cells access the SED during an ongoing response following oral immunization.

We found that some GL7^−^ GFP^+^ B cells in SED were BCL6^+^ (20%) while a majority (70%) expressed this transcription factor in the GC. This observation further supports the notion of a functional role of the activated GL7^−^ B cell population in PP during an ongoing immune response. By contrast, and as expected, a majority of splenic GC GFP^+^ B cells were GL7^+^ (>85%) after an intraperitoneal immunization with NP-CT. Differential expression of GL7 was recently described also among PP T cells within the GC^57^. Thus, the regulatory microenvironment in PPs appeared to be significantly different from that of spleen and, probably, other secondary lymphoid tissues. Of note, the low expression of GL7 in GFP^+^ PP B cells was observed irrespective of the immunization route, the number of immunizations or the source of adoptively transferred naïve B cells, spleen or PPs. The GL7-reactive antibody used to define GC B cells detects α2,6-linked N-acetylneuraminic acid on glycan chains, an epitope highly expressed in GC B cells due to lost expression of CMP-Neu5Ac hydroxylase (CMAH)^43^. Interestingly, in mice that lack *Cmah* gene expression all B cells express high levels of the GL7 epitope, and this is associated with B cells being hyper-responsive to Ig-crosslinking. Therefore, B cell antigen responsiveness may be more tightly regulated in GL7^−^ than GL7^+^ PP B cells and supports the idea that the regulatory microenvironment in PPs is significantly different from that of other secondary lymphoid tissues. For B cells to traffic from the GC to SED and back to the GC we would have to predict that expression of migratory cues needs to shift. For example, CCR6 and CXCR5 could be expressed reciprocally, but may at the same time not be mutually exclusive. In a preliminary single cell RNAseq analysis we found evidence of a possibly alternating dominance of CCR6 and CXCR5 in GFP^+^ B cells from ongoing GC responses in PP (Komban et al., unpublished). Hence, we believe this finding supports that GL7^−^ B cells can shuttle between the GC and SED in the PP, similar to what has been described for splenic marginal zone B cell delivery of antigen to the FDC in the follicle^58,59^. However, the exception being that the splenic marginal zone B cells capture antigen with complement receptors, while in the PP this antigen-transporting system is dependent on specific BCR-recognition of antigen, enabling a highly selective system. In this regard, the PP transport pathway appears more similar to that described for the lung, where specific B cells can transport particulate antigen from the lung to the B cell follicles of the spleen^60^.

The GL7-phenotype was not stable. When GL7^+^ and GL7^−^ cells were adoptively transferred into a “naïve” animal receiving antigen 24 h prior to transfer, we observed in both cases that the cells homed to GC and that 20% expressed GL7. RNAseq analysis showed that the expression of the *Gpr138* (*Ebi2*) gene was higher in GL7^−^ compared to GL7^+^ GFP^+^ B cells. This gene has been found more strongly expressed in B cells migrating to the outer follicular region, and repression of the *Gpr138* gene, required for GC localization, is mediated by an up-regulation of *Bcl6*^61,62^. Hence, higher *Gpr183* gene expression, possibly related to lower *Bcl6* expression, would support a migratory function of GL7^−^ GFP^+^ GC B cells, possibly to the SED. As we found no difference in the expression of *Cxcr4* between GL7^−^ and GL7^+^ GFP^+^ B cells, we concluded that neither population was imminently destined to leave the PP^63^. Rather, GL7^−^ B cells were likely to undergo further differentiation. However, we observed stronger expression of genes associated with memory B cell development, such as *Gpr138*, *Cd38* and *Ccr6*, in GL7^−^ as compared to GL7^+^ GFP^+^ B cells, supporting the idea that the GL7^−^ GFP^+^ GC B cells differentiated towards precursor memory and memory B cells stages^45–47^. Of note, though, since GL7^−^ B cells were found both in LZ and DZ we must conclude that lack of GL7 expression, per se, does not identify precursor memory B cells, exiting the LZ^45,47^. Nevertheless, we propose that the GL7^−^ B cell population in GC host more cells destined to enter a memory stage than GL7^+^ B cells. Whether this constitutes a distinct functional stage or not in the GC need further investigation using single cell RNAseq analysis. However, it appears that antigen transport from SED to the GC is a functional task that is maintained by antigen-specific GL7^−^ B cells during an immune response, which appears to be unique to PPs.

Taken together, this is the first study to provide direct evidence that during an immune response in PP, following oral immunization, antigen-specific B cells in SED interact closely with M cells in the FAE and bind antigen. The novel M cell-B cell antigen transporting pathway in PP could still operate in the CD11c-DTR mice treated with DT, which depleted a majority of DCs, including DCs in SED. In sharp contrast, M cell deficient mice largely failed to show antigen-binding B cells in the SED. The fact that higher frequencies of antigen-binding B cells were observed in SED than GC, and that within 2 h CCR6^−^GL7^+^ GC B cells were also found to carry NP-PE supports that this pathway provides a means to transport antigen from the SED to the the GC. Noteworthy, even after 2 h the GFP^+^ PP B cells had still surface bound antigen. We propose that this way re-utilization of multiple PPs occurs and that the GC reaction can promote the clonal B cell expansion and selection that ultimately leads to a synchronized IgA plasma cell response in the gut lamina propria. Despite that we failed to demonstrate a migration of activated B cells from the GC to SED using photoactivation we would still like to propose that the GL7^−^ B cells emanated from the GC reaction itself and through up-regulated expression of the *Ccr6*-gene and down-modulation of the *Cxcr5* and *Bcl6* genes B cells can move from the GC to SED and, reciprocally, by upregulating the *Cxcr5* and down-modulating the *Ccr6* gene, B cells will return to the GC. We also predict that if this M cell-B cell axis is disrupted in PPs severe reduction in the gut IgA plasma cell response will occur^9,64^.

## Methods

### Mice and immunizations

Permision was granted from the animal ethical comitte in Gothenburg (permit 25/14 and 150/15) and by permit from the Weizmann Institute IACUC committee to perform the experiments. Apart from C57BL/6 mice (Taconics, Bomholt, Denmark), for all NP-specific B cell experiments we used F1 mice generated after crossing C57BL/6 and homozygous B1–8^hi^ GFP mice^23^ (generously provided by M. Nussenzweig, Rockefeller University, New York, NY). Experiments in M cell deficient mice were performed with female RANK^ΔIEC^ (Vil-Cre RANK-flox) and RANK-flox tg-controls RANKF/F mice^9,51^, and DC depletion experiments were done using CD11c-DTR transgenic mice injected intraperitoneally with 4 ng/g body weight of diphteria toxin^54^. PA-GFP, CFP, AID-cre, and Rosa26^fl-stop-fl-tdTomato^ mice were from Jackson laboratory. The mice were bred and housed under specific pathogen-free conditions at the animal facility Experimental Biomedicine (EBM) at the University of Gothenburg or at the Weizmann Institute of Science. Mice aged 6–10 weeks were used in the experiments. NP-specific splenic λ-expressing GFP^+^ B cells were prepared through depletion of non-B cells and κ-expressing B cells using an EasySep Mouse B cell isolation kit (19854, Stem Cell Technologies, Manchester, UK) supplemented with 2 μg anti-mouse κ-chain biotinylated antibody (BD Biosciences, San Jose, CA). To prepare NP-CT, CT was dialyzed in distilled water for one day before mixing it with an equal volume of 0.1  M NaHCO_3_ and 20  mole NP-OSu (Biosearch Technologies, Novato, CA) per mole CT. The mixture was incubated for 12 h at RT and transferred into a Slide-A-Lyzer dialysis cassette and first dialyzed against 0.05 M NaHCO_3_, followed by water before the protein concentration was determined using a BCA assay (23225, Thermo Fisher Scientific, Rockford, IL). Mice were immunized with 10 μg NP-CT per orally (p.o.) or 2 μg NP-CT intraperitoneally (i.p.).

### Ligated loop assay

C57BL/6 wild-type mice, M cell deficient or proficient littermates or CD11c-DTR transgenic mice were adoptively transferred with NP-specific splenic GFP^+^ B cells followed by an oral immunization with NP-CT and 10 days later the mice were anesthetized using isoflurane and an incision was made along *linea alba* of the abdomen to expose the intestine. Diphteria toxin was delivered to CD11c-DTR mice on day 9. PPs were identified and ligated loops of intestine were made according to the protocol^65^. Briefly, the ligated loops were 1.5–2 cm long and were injected with 50 µl of PE (N-5070–1-BS, LGC Biosearch tech) or NP-PE at a concentration of 3.3 mg/ml. The gut was punctured outside of the loop and the ligation tightened after injection to avoid any contamination from NP-PE during injection After ligation and injection of antigen into the loops the abdomen was closed with sutures and the mice were kept under anesthesia for 1 or 2 h. For control purposes mice were sacrificed after 5 min to analyze antigen-leakiness. To study antigen-binding to B cells, PPs were excised after 1 and 2 h and single cell suspensions were prepared for FACS analysis and tissues were also taken for sectioning and further microscopic analysis.

### Immunohistochemistry

The mice were killed and the spleen, PP, or LP (lamina propria) were embedded in TissueTek OCT compound and snap frozen in liquid nitrogen. Tissues were fixed in 4% paraformaldehyde and 10% sucrose for 1 h before transferring to 30% sucrose solution and kept at 4 °C overnight. Frozen sections (7–9 μm) were fixed in 100% acetone and blocked with 5% normal horse serum in PBS for 15 min. Antibodies used to stain sections were anti-mouse GL7-eflour 660 (1:200, 50–5902–82, eBiosciences), Ki67-eflour 450 (1:200, 45–5698–80, eBiosciences), CXCR4-V450 (1:100, 560875, BD Bioscience), CD83-PE (1:200, 12–0831–82, eBiosciences), CD4-PerCp (1:300, 100431, Biolegend), CD11C-BV421 (1:100, 562782, BD Bioscience), EpCam-APC (1:50, 118214, Biolegend), CCR6-BV421 (1:50, 564736, BD Bioscience), CD138-PE (1:100, 553714, BD Bioscience), BCL6-A647 (1:50, 561524, BD Bioscience), CD35-Biotin (1:200, 553816, BD Bioscience), B220-eflour 450 (1:400, 48–0452–82, eBiosciences), B220-biotin (1:500, 553086, BD Bioscience), followed by Streptavidin-Alexa Flour 594 (1:150, 511227, Life Technologies). Anti-GP2 Biotin (1:100, D278–6, MBL) followed by Streptavidin-Alexa Flour 594 (1:150, 511227, Life Technologies) and anti-IgA-biotin (1:100, 556978, BD Bioscience) or anti-Ephrin B1-biotin (1:200, BAF473, R&D Systems, Minneapolis, MN) followed by Streptavidin-Alexa Flour 647 (1:150, S32357, Life Technologies) were used. Microscopy was performed at the Center for Cellular Imaging using the confocal Zeiss LSM 700 inverted system and LSM software (Carl Zeiss, Oberkochen, Germany).

### Flow cytometry and cell sorting

Lymphocytes were isolated as described^66^ and stained with anti-mouse CD19-PECy7 (1:300, 25–0193–82, eBiosciences), IgD-PerCPCy5.5 (1:400, 405710, Biolegend), GL7-eFluor 450 (1:200, 48–5902–82, eBiosciences), or A647 (1:200, 53–5902–80, eBiosciences). For studying the phenotypic expression of GFP^+^ B cells in the germinal center, the following anti-mouse antibodies  were used: CD1d-PE (1:200, 12–0011–81, eBiosciences), CD16-PE (1:200, 561727, BD Bioscience), CD23-PE (1:200, 12–0232–81, eBiosciences), CD24-PE (1:200, 561079, BD Bioscience), CD44-PE (1:200, 553134, BD Bioscience), CD54-PE (1:200, 553253, BD Bioscience), CD69-PE (1:200, 553237, BD Bioscience), CD62L-PE (1:200, 12–0621–81, eBiosciences), CD80-PE (1:200, 553769, BD Bioscience), CD86-PE (1:200, 561963, BD Bioscience), CD95-PE (1:200, 554258, BD Bioscience), MHCII-AlexaFlour700 (1:200, 56321–80, eBiosciences), were analysed using LSR II (BD Biosciences) flow cytometer. For, dark and light zone determination, anti-CXCR4-V450 (1:200, 560875, BD Bioscience) and anti-CD83-PE (1:200, 12–0831–82, eBiosciences) were used along with other germinal center markers. To  confirm a germinal center phenotype BCL6-PE (1:50, 561522, BD Bioscience), was used. CCR6-PE (1:75, 557976, BD Bioscience) was used to identify the SED population. After labeling, cells were washed twice and analyzed or sorted using an LSR II (BD Biosciences) or a FACS-Aria III (BD Biosciences). Data analysis was performed using FlowJo software (Tree Star). Live gates were set on lymphocytes by forward and side scatter, and singlets identified using forward scatter height and area plots.

### RNAseq analysis

FACS-sorted GFP^+^ and GFP^−^ B cell single cells populations were prepared from spleen or PP and submerged in 350 µl RLT buffer (Qiagen, Hilden, Germany). The tissue was disrupted and homogenized and RNA extracted using RNeasy micro kits (74004, Qiagen). RNA was sent to BGI for transcript quantification using RNA sequencing following amplification using a SMARTer PCR cDNA Synthesis Kit (Takara Biomedical Technology, Beijing, China). The sequencing data was analyzed using CLC Genomic Workbench version 11.0.1 (Qiagen). After importing paired sequencing data, the data was aligned to mouse genome GRCm38 using the RNA-Seq analysis workflow. Gene-expression tracks (GE) were used for the generation of PCA plots. For the generation of Venn diagrams and supplementary table 2, genes differentially expressed in the groups were identified using the Differential Expression for RNASeq tool, and the Venn diagrams were constructed based on genes with a fold change > 1.5 and FDR < 0.05. Genes used for the gene ontology (GO) analysis was filtered using the same settings within CLC Gneomic Workbench. For the gene heat map and clustering, genes differentially expressed in the different groups were identified using the Differential Expression for RNASeq tool and the clustering was then based on genes that showed a fold change > 1.5 and FDR < 0.5.

### In vivo imaging

Mice were anesthetized with a mixture of 50 mg ketamine, 15 mg xylazine, and 2.5 mg acepromazine per kg of body weight and were maintained under anesthesia by inhalation of 1% isofluorane. One gut loop, with one PP was surgically exposed and immobilized, covered with a coverslip and placed under the microscope objective. Zeiss LSM 880 upright microscope combined with Coherent Chameleon Vision laser was used for in vivo photoactivation and imaging experiments. Pictures were acquired with a femtosecond-pulsed two-photon laser tuned to 940 nm. Filter cube contained 565 LPXR to split the emission to a PMT detector (with a 579–631 nm filter for dsRed and TdTomato fluorescence) and to additional 505 LPXR mirror to split the emission to 2 GaAsP detectors (with a 500–550 nm filter for GFP fluorescence and a 460–480 nm filter for CFP fluorescence). PAGFP^+^ B1–8^hi^ B cells were photoactivated in the SED compartment. After 1–4 h, cells were counted in and outside the SED compartment and the proportion of PA-GFP^+^ cells outside the SED (outside the SED/SED) was calculated. SED or GCs in PP were photoactivated at 820 nm^49,50,67^. Tile images were acquired as 100–150 µm *Z*-stacks with 5 µm steps between each *Z*-plane. The zoom was set to 1.2, and pictures were acquired at 512 × 512 *x*-*y* resolution.

### Statistical analysis

All the graph plotting and statistical analyses were performed using the GraphPad Prsim 6.0 software (GraphPad Software Inc., San Diego, CA, USA). Values are given ± s.d. and the number of experiments and group sizes are given in the Figure legends. Error bars represent values of s.d. The *p* values < 0.05 were considered to indicate statistical significance (not significant (NS), *p < 0.05, **p < 0.01, ***p < 0.001, and ****P ≤ 0.001.

### Reporting summary

Further information on research design is available in the Nature Research Reporting Summary linked to this article.

## Supplementary information

Supplementary Information

Reporting Summary

Description of Additional Supplementary Files

Supplementary Data 1

Supplementary Data 2

Source Data

## Data Availability

The RNASeq data have been deposited in the NCBI Gene expression Omnibus (GEO) under the accession code GSE129103 and the sequence files in the NCBI Sequence Read Archive under the accession code SRP189986. Source data for the figures are included in the Source Data file. Further information’s and requests for resources should be directed to and will be fulfilled by Nils Lycke.
