# Supplementary material for: Activated Peyer′s patch B cells sample antigen directly from M cells in the subepithelial dome
Source: Nat Commun. 2019 Jun 3;10:2423. doi: 10.1038/s41467-019-10144-w (PMC6547658; doi:10.1038/s41467-019-10144-w)
Supplement: Supplementary file 1 — Supplementary Information [file 41467_2019_10144_MOESM1_ESM.pdf]

Supplementary information

Activated Peyer´s patch B cells sample antigen directly from M cells in the subepithelial dome

Komban et al.

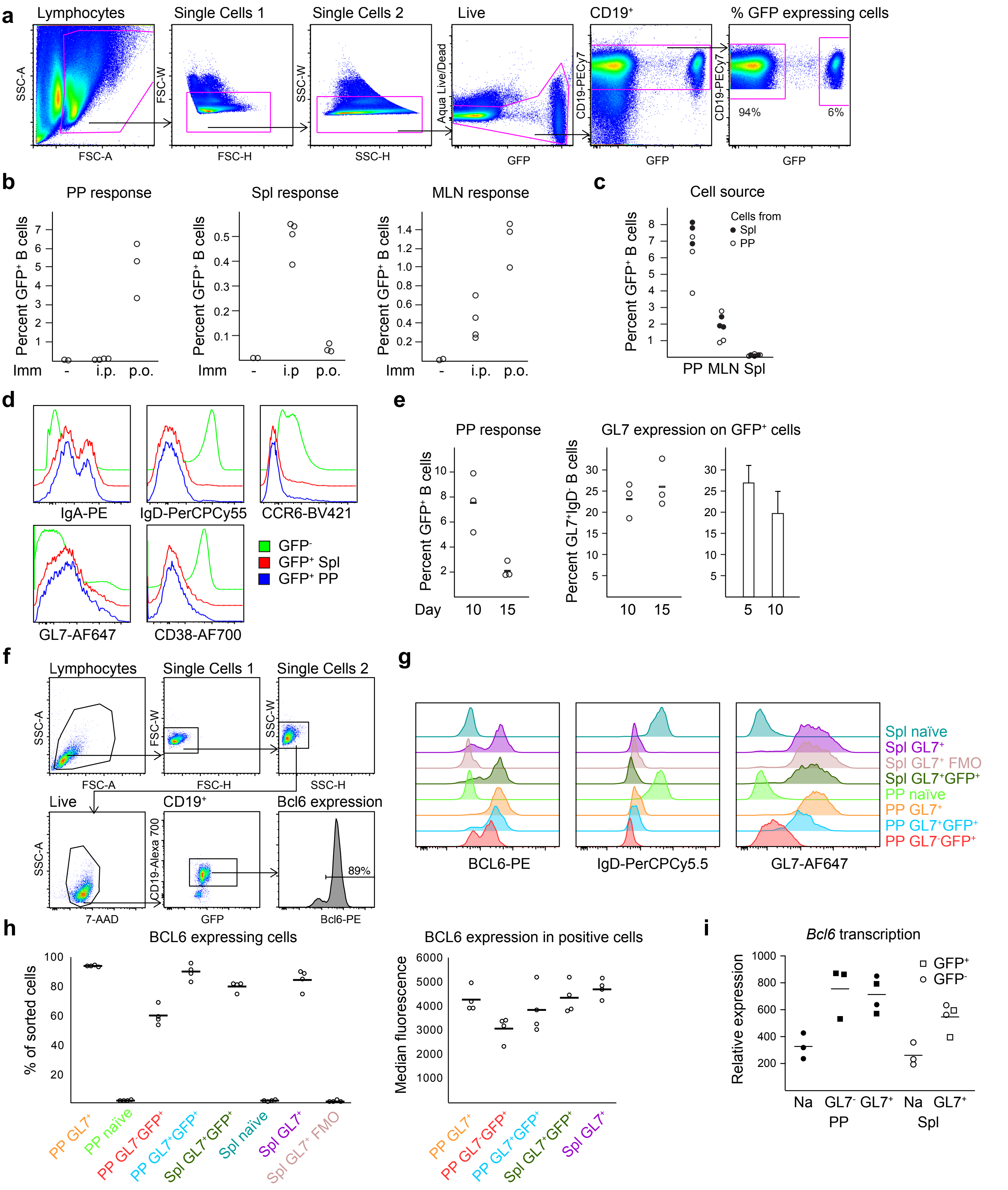

Supplementary Figure 1: Oral immunization with NP-CT triggers a PP germinal center (GC) response where few cells express GL7.
a Flow cytometric analysis used in panels Fig 1b, 2c and Supplementary Fig 1a-e, 4c to identify GFP+ and GFP- B cells in mice transfered with NP-specific κ-depleted B cells from B1-8hi/GFP mice. **b** Mice were immunized intraperitoneally (i.p.) or perorally (p.o.) with NP-CT one day after transfer and on day ten following immunization the proportion of GFP expressing B cells was determined in Peyer´s patches (PP), spleen (Spl) or messenteric lymph nodes (MLN) was determined. Only p.o. immunization resulted in a PP response, i.p. immunization triggered a much stronger splenic response, whereas both immunization routes triggered MLN responses. **c,d** Mice were transferred with B cells from Spl or PP before immunization. No difference was evident in the frequency of responding cells depending on the origin of the donor cells, and the cells did not show differences in expression of markers depending on the origin of the cells. **e** After p.o. immunization the response peaked at day 10, but the frequency of IgD-GL7+ GC phenotype cells was similar all through the response. **f** Flow cytometric analysis of sorted cells intracellularly stained to detect BCL6 expression. **g,h** Mice that had received B1-8hi/GFP splenic B cells were Mice were simultaneously p.o. and i.p. immunized with NP-CT one day after transfer, and on day ten cells from PP and Spl were isolated using flow cytometric cell sorting before fixation, permeabilization and staining with an anti-BCL6 antibody. **g** Flow cytometric analysis of one representative mouse comparing the expression of BCL6, IgD and GL7 in cell populations from Spl and PP and a Spl GL7+ FMO control with no anti-BCL6 antibody added. **h** To the left are shown the percentage of cells expressing BCL6 in the populations and to the right the median fluorescence (MFI) for BCL6 staining in positive cells. **i** RNA expression of *Bcl6* in the indicated sorted populations from PP and Spl based on the RNASeq datasets used in Figure 3 but with Spl GL7+ cells also included; Na = naïve GL7- B cells). Source data are provided as a Source Data file.

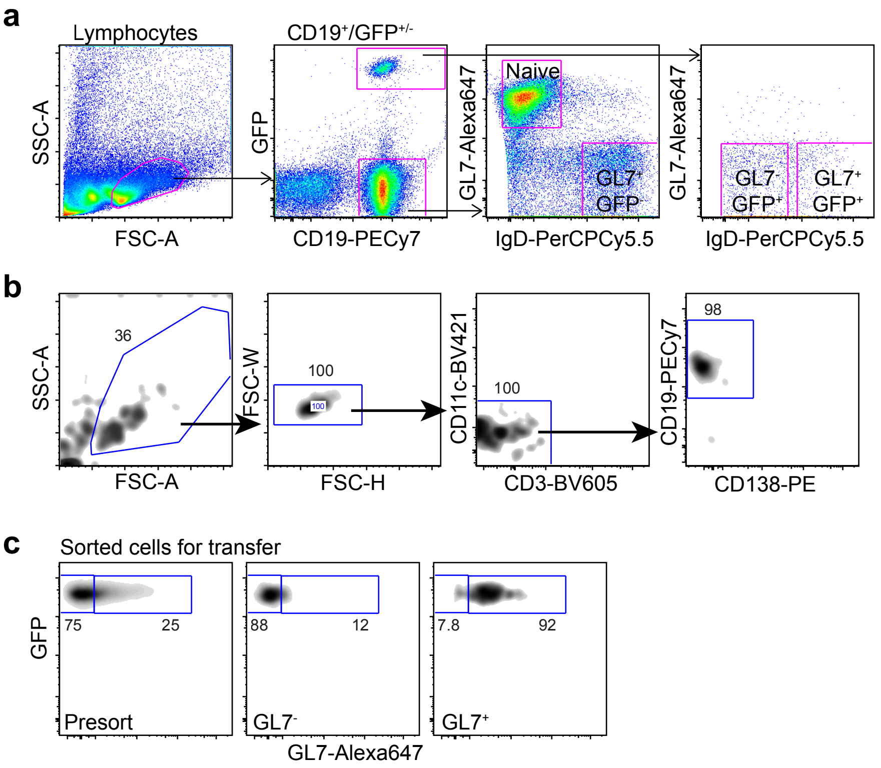

Supplementary Figure 2: Gating of cell populations in Fig 2b-d, and for flow cytometric cell sorting and purity determination of GL7^{+} and GL7^{-} fractions. **a** Gating strategy for the analysis of different cell populations analysed in Fig. 2b-d and g. **b** Gates used to sort and analyse purity of GL7^{+} and GL7^{-} activated GFP^{+} B cells isolated from PP. The example shows the purity of a GL7^{+}GFP^{+} cells after sorting. The gating strategy was used for sorting cells for transfer in Fig 2 f-h and for RNASeq analysis in Figure 3. **c** Analysis of purity of sorted GL7^{+} and GL7^{-} fractions from mice that had been transferred with NP-specific cells and orally immunized with NP-CT 10 day before. The left plot shows the expression of GL7 on the B cells before sorting, and the two right plots the purity of the GL7^{+} and GL7^{-} fractions after sorting. The purified populations were subsequently transferred into mice that had been orally immunized one day before the transfer.

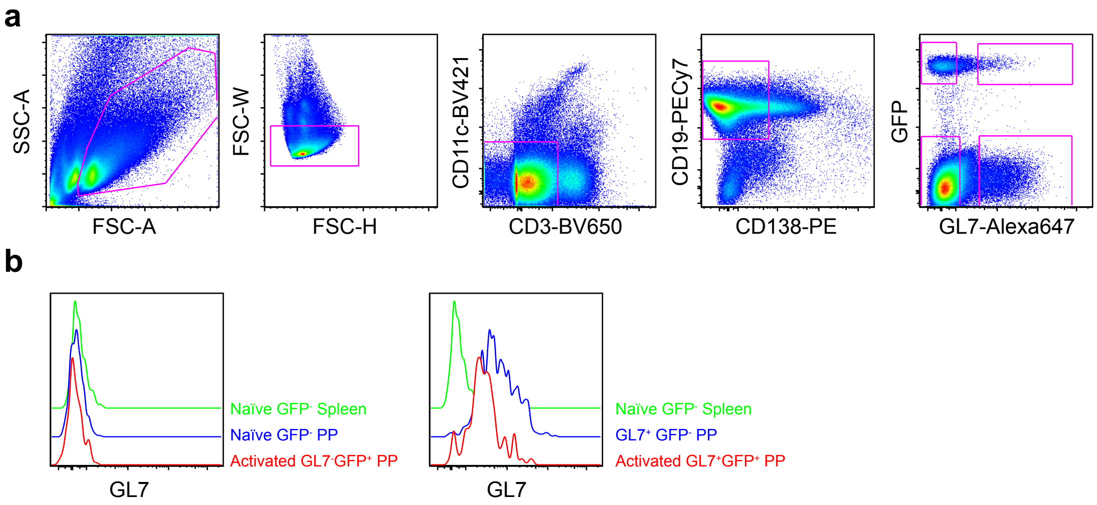

Supplementary Figure 3 : Sorting and purity of cell fractions used for RNASeq.
a Activated GL7$^+$ and GL7$^-$ NP-specific GFP$^+$ PP B cells were isolated using flow cytometric cell sorting ten days after oral immunization with NP-CT and naïve GL7$^-$ B cells and naturally activated GL7$^+$ from PP from nonmanipulated mice. The gating structure for isolation of all four populations are shown although they were never purified from the same mouse. **b** Before preparation of RNA for RNASeq analysis, the expression of GL7 are confirmed for the sorted populations, with the expression of GL7 on sorted naïve splenic cells as a comparison. The left plot shows naïve PP cells and activated GL7$^-$ cells, and the right NP-CT and spontaneously activated GL7$^+$ PP cells.

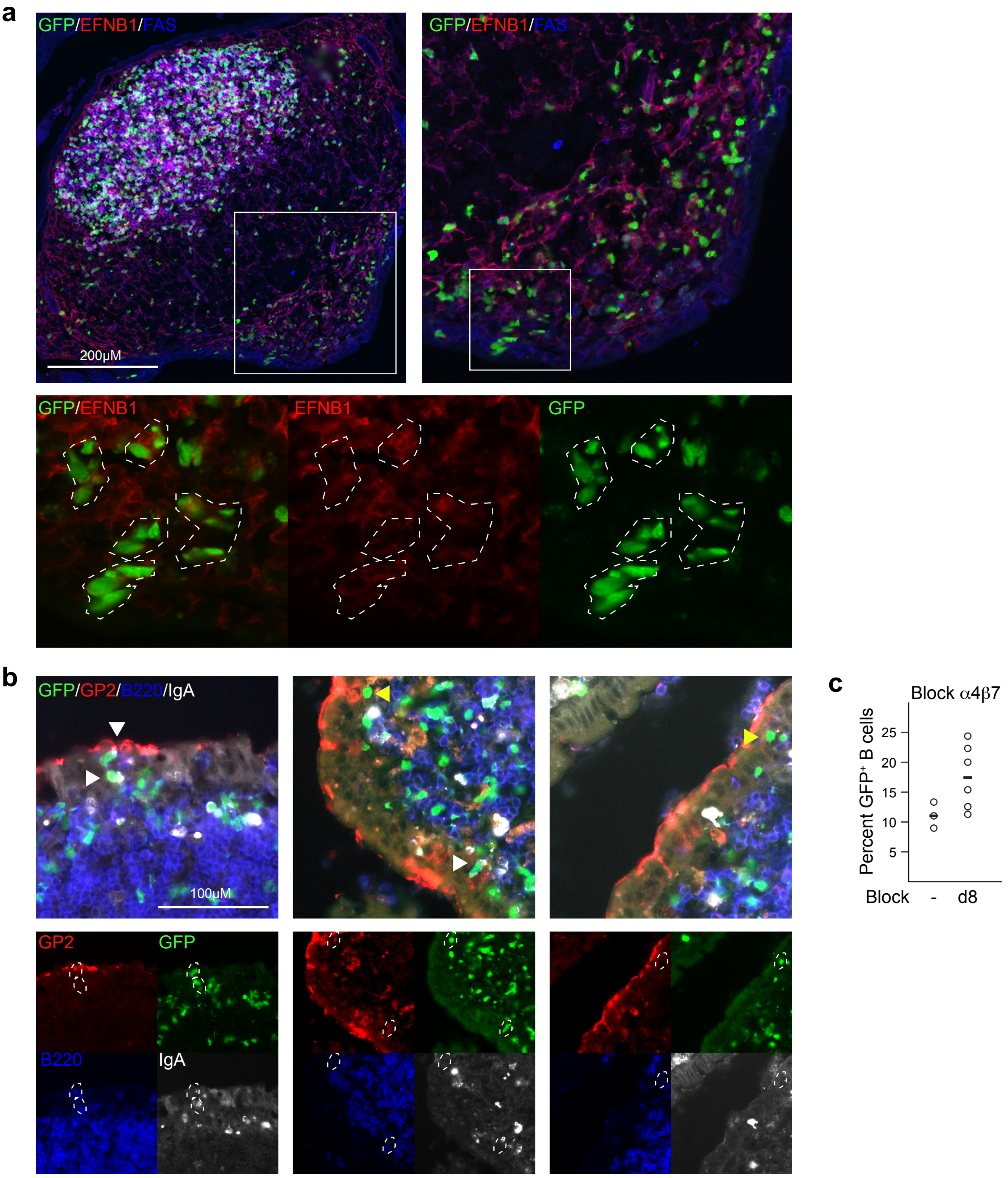

Supplementary Figure 4 : Microscopy of antigen-specific B cells in the and requirement for entry
a A confocal microscopy image of a PP from a mouse transferred with GFP-expressing NP-specific B1-8^{hi} B cells that had been orally immunized with NP-CT ten days before the tissue was isolated, prepared and stained with the indicated antibodies. GFP-expressing B cells are present both in the GC and the SED region. EFNB1 and FAS was expressed at high levels in the GC (left), and close-ups of the SED region (right) demonstrated that EFNB1 and FAS was co-expressed (violet) on cells also in this region although the expression was lower. Analysis of individual chanels demonstrated cell surface expression of EFNB1 on GFP-expressing cells. **b** Sections from the SED regions prepared and analyzed as above demonstrated that IgA expressing (white arrowheads) and non-expressing (yellow arrowheads) antigen-specific B cells interacted closely with GP2 expressing M cells in the follicle associated epithelium. c Blocking the LPAM ($\alpha4\beta7$) integrin at day eight of the response slightly increased the number of GFP expressing cells in PP at day ten, arguig for that that few cells enter from the circulation during a response and that most B cells in a PP expand within that PP during a response. Source data are provided as a Source Data file.

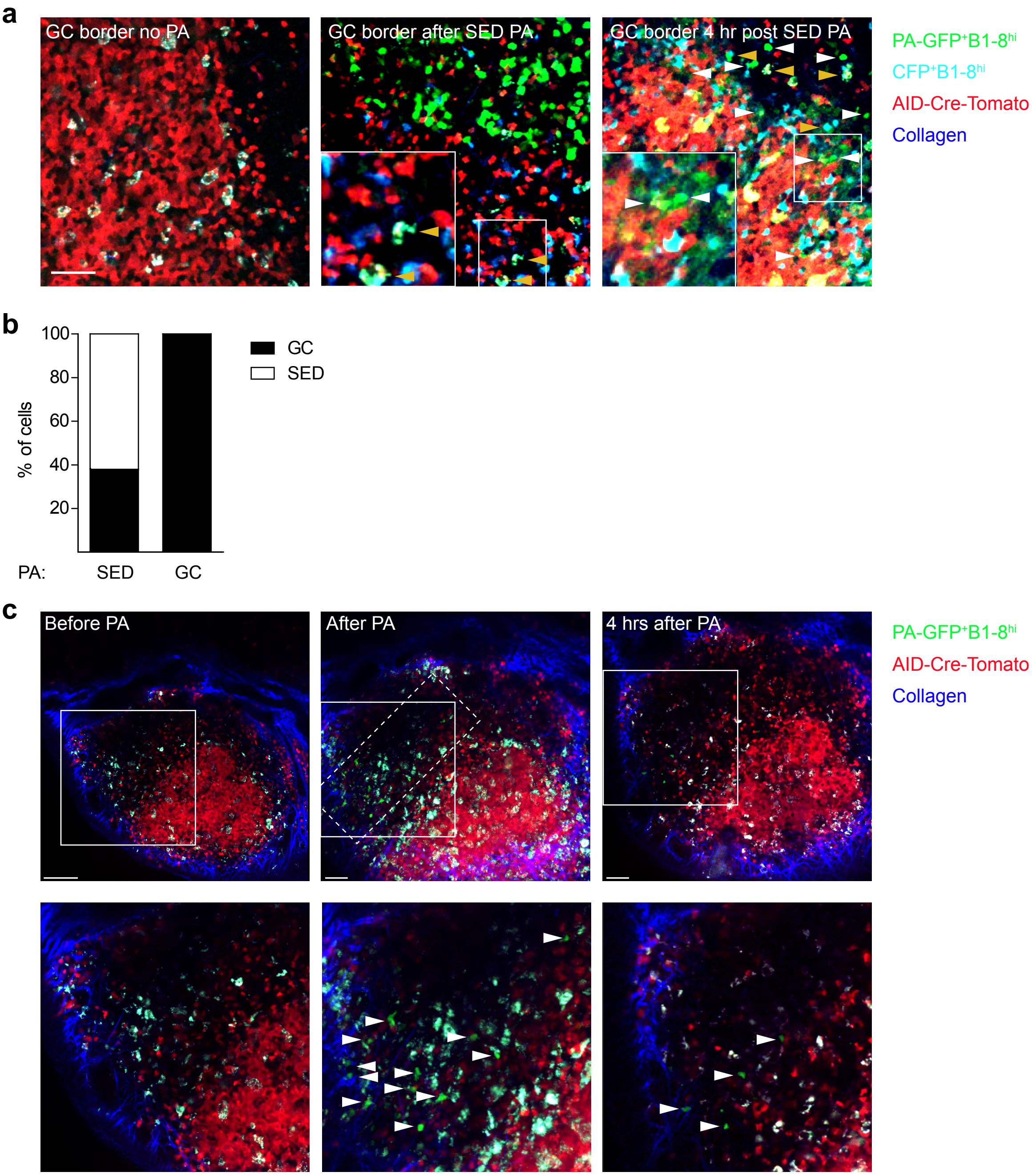

Supplementary Figure 5 : Limited autofluorescence is in macrophages and photoactivated signal in B cells in PP GC and SED
a Close up figures of the GC border area of mice immunized with NP-CT ten days before analysis showing the tissue before photoactivation (PA) , directly after PA of the SED region and four hours after SED PA. No GFP expression was evident close to or within the GC area before PA. Macrophages were detected in the CFP channel due to autoflourescence but also low levels of GFP were evident at the GC border directly after SED activation (marked with orange arrowheads). However, four hours after PA, B cells that only expressed GFP were also evident in the area (marked with white arrowheads) as well as smaller numbers of macrophages expressing both markers (marked with orange arrowheads). **b** Cells expressing GFP were individually counted based on if they were in the GC area or in the SED area four hours after activation (regardless of the photoactivation area). When cells were activated in the SED are, cells outside of the photoactivation area moved towards the GC. No similar movement from GC to SED could be detected and all cells outside of the photoactivation area were still within the GC after GC photoactivation. **c** A SED PA experiment was performed in the absence of guiding CFP cells. In this experiment, few B cells were activated due to that the SED region could not be correctly identifyed during activation. No GPF staining could be detected in the GC area just after photoactivation although autoflourescent macrophages were detected. Four hours after photoactivation, the number of GFP^{+} cells in the region outside of the GC had diminished similarly to what we observed when SED had been targeted using CFP expressing B cells. Scale bars = 80μm.

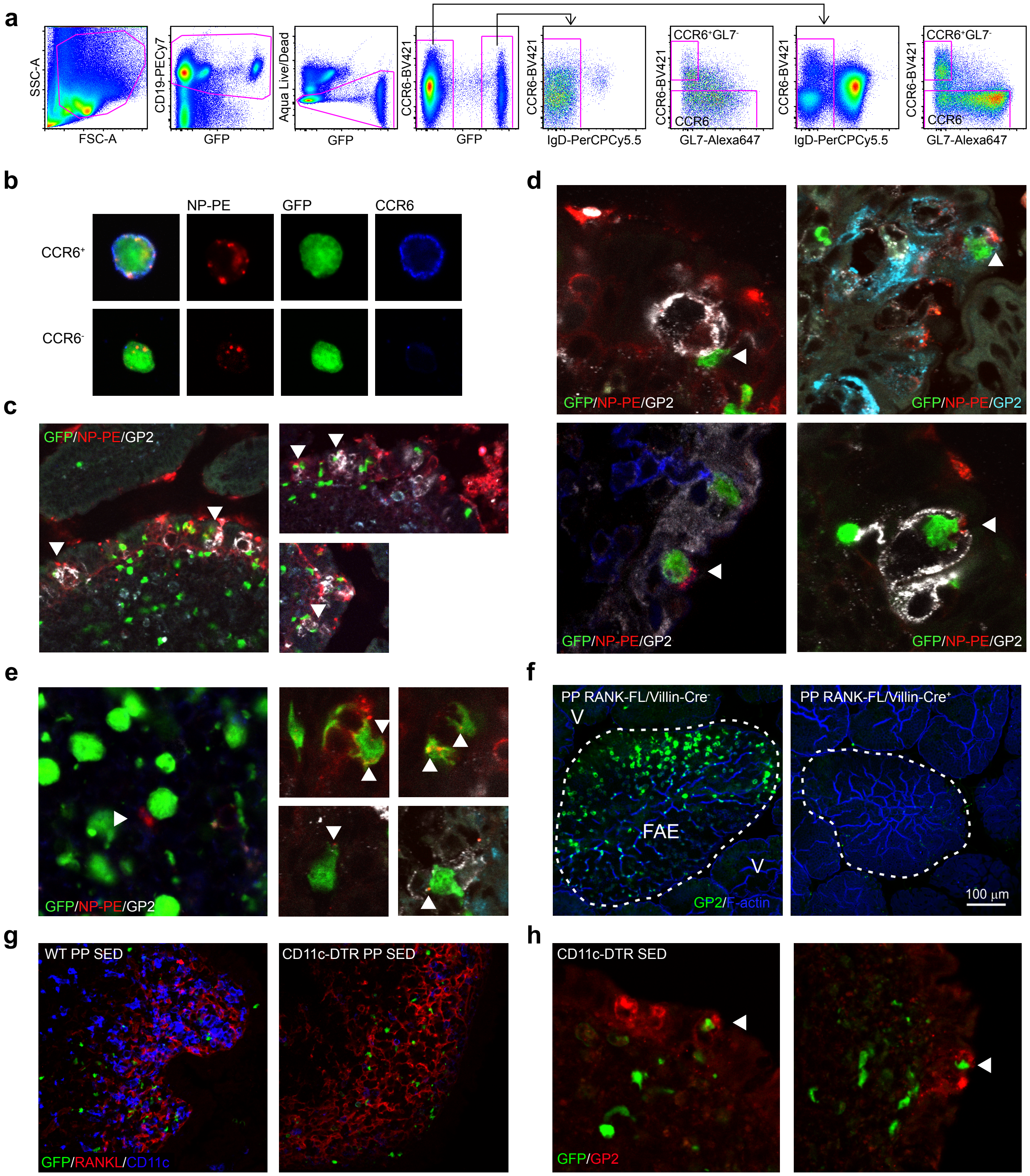

Supplementary Figure 6 : Close interactions between NP-PE, M cells and B cells in the SED region
a Flow cytometric analysis of NP-PE binding in panels Fig 6c-e, j, l and m of PP cells from instestinal loops injected with NP-PE. Single live CD19-expressing B cells were divided into two based on GFP expression and non-naïve IgD-negative cells were divided into $GL7^-CCR6^+$ SED and $CCR6^-$ GC cells before analysis of NP-PE binding. **b** Confocal microscopy of NP-PE binding GFP^+ cells fixed directly after flow cytometric cell sorting, demonstrating that the NP-PE antigen was primarily carried at the cell surface on $GL7^-CCR6^+$ and $CCR6^-$ B cells. **c-e** Mice were immunized and loops injected with NP-CT as described in Fig. 6 and sections were prepared for confocal microscopy. **c,d** Close interaction between B cells (marked with white arrowheads) and M cell and NP-PE antigen was observed. **e** Close ups of B cells carrying antigen on the cells surface (indicated by white arrowheads) that are not in direct contact with M cells. **f** Confocal microscopy demonstrates a total absence of GP2 expressing M cells in the follicle associated epithelium overlaying the PP in RANK-FL/Villin-Cre^+ mice used in Fig. 6f. **g** Microscopy demonstrates an essentially complete depletion of CD11c-expressing cells in the SED region 24 hours after diphtheria toxin treatment of CD11c-DTR mice used in Fig. 6g. **h** Close interactions between M cells and antigen-specific B cells are still evident after depletion of CD11c-expressing cells from the SED.
