# Supplementary material for: Activated Peyer′s patch B cells sample antigen directly from M cells in the subepithelial dome
Source: Nat Commun. 2019 Jun 3;10:2423. doi: 10.1038/s41467-019-10144-w (PMC6547658; doi:10.1038/s41467-019-10144-w)
Supplement: Supplementary file 2 — Reporting Summary [file 41467_2019_10144_MOESM2_ESM.pdf]

nature research

Corresponding author(s): Nils Lycke, Mats Bemark

Last updated by author(s): 2019/04/11

Reporting Summary

Nature Research wishes to improve the reproducibility of the work that we publish. This form provides structure for consistency and transparency in reporting. For further information on Nature Research policies, see Authors & Referees and the Editorial Policy Checklist.

Statistics

For all statistical analyses, confirm that the following items are present in the figure legend, table legend, main text, or Methods section.

n/a	Confirmed	
☐	☒	The exact sample size (n) for each experimental group/condition, given as a discrete number and unit of measurement
☐	☒	A statement on whether measurements were taken from distinct samples or whether the same sample was measured repeatedly
☐	☒	The statistical test(s) used AND whether they are one- or two-sided<br>*Only common tests should be described solely by name; describe more complex techniques in the Methods section.*
☒	☐	A description of all covariates tested
☒	☐	A description of any assumptions or corrections, such as tests of normality and adjustment for multiple comparisons
☒	☐	A full description of the statistical parameters including central tendency (e.g. means) or other basic estimates (e.g. regression coefficient) AND variation (e.g. standard deviation) or associated estimates of uncertainty (e.g. confidence intervals)
☒	☐	For null hypothesis testing, the test statistic (e.g. F, t, r) with confidence intervals, effect sizes, degrees of freedom and P value noted<br>*Give P values as exact values whenever suitable.*
☒	☐	For Bayesian analysis, information on the choice of priors and Markov chain Monte Carlo settings
☒	☐	For hierarchical and complex designs, identification of the appropriate level for tests and full reporting of outcomes
☒	☐	Estimates of effect sizes (e.g. Cohen's d, Pearson's r), indicating how they were calculated

Our web collection on statistics for biologists contains articles on many of the points above.

Software and code

Policy information about availability of computer code

Data collection	FACSDiva software for collection of flow cytometric data, LSM software for confocal microscopy, Graphpad Prism for statistical analysis, Zeiss ZEN microscope software for PA experiments
Data analysis	FlowJo version 9.9.6 (FlowJo LLC) , CLC genomic workbench version 11.0.1 (Qiagen), ImageJ2 2.0.0-rc-64 for Java 8 running through Fiji, Imaris for image analysis of photoactivation

For manuscripts utilizing custom algorithms or software that are central to the research but not yet described in published literature, software must be made available to editors/reviewers. We strongly encourage code deposition in a community repository (e.g. GitHub). See the Nature Research guidelines for submitting code & software for further information.

Data

Policy information about availability of data

All manuscripts must include a data availability statement. This statement should provide the following information, where applicable:

- Accession codes, unique identifiers, or web links for publicly available datasets
- A list of figures that have associated raw data
- A description of any restrictions on data availability

RNASeq data is available through NCBI GEO GSE129103 and NCBI Sequence Read Archive (SRA) SRP189986, other data through contact with the corresponding authors.

Field-specific reporting

Please select the one below that is the best fit for your research. If you are not sure, read the appropriate sections before making your selection.

☒ Life sciences ☐ Behavioural & social sciences ☐ Ecological, evolutionary & environmental sciences

For a reference copy of the document with all sections, see nature.com/documents/nr-reporting-summary-flat.pdf

Life sciences study design

All studies must disclose on these points even when the disclosure is negative.

Sample size	No sample-size calculations were performed. Sample size was determined to be adequate based on the magnitude and consistency of measurable differences between groups. To avoid unethical use of mice, we determined the number of animal use according to field standards and reproducibility achieved among different mice.
Data exclusions	No data excluded
Replication	Replicate experiments were successful. Each experiment was controlled by technical and biological replicates as indicated in figure legends.
Randomization	Mice were assigned according to their genotype. Litter mates and sex-matched animals were used whenever possible. All other parameters are random.
Blinding	No blinding of data performed

Reporting for specific materials, systems and methods

We require information from authors about some types of materials, experimental systems and methods used in many studies. Here, indicate whether each material, system or method listed is relevant to your study. If you are not sure if a list item applies to your research, read the appropriate section before selecting a response.

Materials & experimental systems

n/a	Involved in the study	
☐	☒	Antibodies
☒	☐	Eukaryotic cell lines
☒	☐	Palaeontology
☐	☒	Animals and other organisms
☒	☐	Human research participants
☒	☐	Clinical data

Methods

n/a	Involved in the study	
☒	☐	ChIP-seq
☐	☒	Flow cytometry
☒	☐	MRI-based neuroimaging

Antibodies

Antibodies used	For microscopy: GL7-eflour 660 (1:200, 50-5902-82, eBiosciences), Ki67-eflour 450 (1:200, 45-5698-80, eBiosciences), CXCR4-V450 (1:100, 560875, BD Bioscience), CD83-PE (1:200, 12-0831-82, eBiosciences), CD4-PerCp (1:300, 100431, Biolegend), CD11C-BV421 (1:100, 562782, BD Bioscience), EpCam-APC (1:50, 118214, Biolegend), CCR6-BV421 (1:50, 564736, BD Bioscience), CD138-PE (1:100, 553714, BD Bioscience), Bcl6-A647 (1:50, 561524, BD Bioscience), CD35-Biotin (1:200, 553816, BD Bioscience), B220-eflour 450 (1:400, 48-0452-82, eBiosciences), B220-biotin (1:500, 553086, BD Bioscience) and GP-2 Biotin (1:100, D278-6, MBL) followed by Streptavidin-Alexa Flour 594 (1:150, 511227, Life Technologies), IgA-biotin (1:100, 556978, BD Bioscience) or Ephrin B1-biotin (1:200, BAF473, R&D Systems, Minneapolis, MN) followed by Streptavidin-Alexa Flour 647 (1:150, S32357, Life Technologies).<br>For flow cytometry: CD19-PeCy7 (1:300, 25-0193-82, eBiosciences), IgD-PerCP-Cy5.5 (1:400, 405710, Biolegend), GL7-eFluor 450 (1:200, 48-5902-82, eBiosciences) and AF647 (1:200, 53-5902-80, eBiosciences), CD1d-PE (1:200, 12-0011-81, eBiosciences), CD16-PE (1:200, 561727, BD Bioscience), CD23-PE (1:200, 12-0232-81, eBiosciences), CD24-PE (1:200, 561079, BD Bioscience), CD44-PE (1:200, 553134, BD Bioscience), CD54-PE (1:200, 553253, BD Bioscience), CD69-PE (1:200, 553237, BD Bioscience), CD62L-PE (1:200, 12-0621-81, eBiosciences), CD80-PE (1:200, 553769, BD Bioscience), CD86-PE (1:200, 561963, BD Bioscience), CD95-PE (1:200, 554258, BD Bioscience), MHCII-A700 (1:200, 56321-80, eBiosciences), CXCR4-V450 (1:200, 560875, BD Bioscience), CD83-PE (1:200, 12-0831-82, eBiosciences), Bcl6-PE (1:50, 561522, BD Bioscience), CCR6-BV421 (1:75, 557976, BD Bioscience) or BV421 (1:100, 140706, BD Bioscience).
Validation	Antibodies specificity was validated according to the manufacturer's instructions and all antibodies used are common and are largely described in the literature.

Animals and other organisms

Policy information about studies involving animals; ARRIVE guidelines recommended for reporting animal research

Laboratory animals	C57BL/6 mice or gene targeted mice on a C57BL/6 background of both sexes were used
Wild animals	Not used
Field-collected samples	Not used
Ethics oversight	Ethical permits for the experiments have been obtained from the animal research ethical committee in Gothenburg and the Weizmann Institute IACUC committee

Note that full information on the approval of the study protocol must also be provided in the manuscript.

Flow Cytometry

Plots

Confirm that:

☒ The axis labels state the marker and fluorochrome used (e.g. CD4-FITC).

☒ The axis scales are clearly visible. Include numbers along axes only for bottom left plot of group (a 'group' is an analysis of identical markers).

☒ All plots are contour plots with outliers or pseudocolor plots.

☒ A numerical value for number of cells or percentage (with statistics) is provided.

Methodology

Sample preparation	Cells were isolated from dissected PP, MLN and spleen using cell strainer nets and syringe plungers before being incubated with antibodies as indicated.
Instrument	Data was collected using BD LSR II or BD Fortessa flow cytometers, Sorting was performed on a BD FACSAria III sorter
Software	Collection was performed using FACSDiva software, analysis using FlowJo version 9.6 software
Cell population abundance	For flow cytometry, typical data are shown for percentage of antigen specific GFP+ cells in transferred mice that has been orally immunized, in general 1-3% of all B cells. Numbers given and example data shown in Supplementary figures for sorted cells.
Gating strategy	Gating performed as indicated in the Figures for antigens which do not have a clear bimodal expression pattern.

☒ Tick this box to confirm that a figure exemplifying the gating strategy is provided in the Supplementary Information.
