# Supplementary material for: Activated Peyer′s patch B cells sample antigen directly from M cells in the subepithelial dome
Source: Nat Commun. 2019 Jun 3;10:2423. doi: 10.1038/s41467-019-10144-w (PMC6547658; doi:10.1038/s41467-019-10144-w)
Supplement: Supplementary file 3 — Description of Additional Supplementary Files [file 41467_2019_10144_MOESM3_ESM.pdf]

## Description of Additional Supplementary Files

Supplementary Data 1

Description: RNAseq profiling: gene expression

Supplementary Data 2

Description: RNAseq profiling: gene expression
